# The utility of physiotherapy assessments delivered by telehealth: A systematic review

**DOI:** 10.7189/jogh.11.04072

**Published:** 2021-12-18

**Authors:** Cherie Zischke, Vinicius Simas, Wayne Hing, Nikki Milne, Alicia Spittle, Rodney Pope

**Affiliations:** 1 Faculty of Health Sciences and Medicine, Bond Institute of Health and Sport, Bond University, Robina, Queensland, Australia; 2 School of Allied Health, Exercise and Sports Sciences, Charles Sturt University, Port Macquarie, New South Wales, Australia; 3 Faculty of Medicine, Dentistry and Health Sciences, The University of Melbourne, Melbourne, Victoria, Australia; 4 School of Allied Health, Exercise and Sports Sciences, Charles Sturt University, Albury, New South Wales, Australia

## Abstract

**Background:**

Telehealth use is increasing due to its ability to overcome service access barriers and provide continued care when disease transmission is of concern. However, little is known of the validity, reliability and utility of performing physiotherapy assessments using synchronous forms of telehealth across all physiotherapy practice areas. The aim of this systematic review was to determine the current clinometric value of performing physiotherapy assessments using synchronous forms of telehealth across all areas of physiotherapy practice.

**Methods:**

A comprehensive search of databases (PubMed/MEDLINE, The Cochrane Library, Embase and EBSCO) was undertaken to identify studies investigating the clinometric value of performing physiotherapy assessments using synchronous forms of telehealth across all physiotherapy practice areas. Following selection, a quality appraisal was conducted using the Brink and Louw or Mixed Methods Appraisal Tool. Evidence regarding validity, reliability and utility of synchronous telehealth physiotherapy assessments was extracted and synthesised using a critical narrative approach.

**Results:**

Thirty-nine studies conducted in a variety of simulated (n = 15) or real-world telehealth environments (n = 24), were included. The quality of the validity, reliability and utility studies varied. Assessments including range of movement, muscle strength, endurance, pain, special orthopaedic tests (shoulder/elbow), Berg Balance Scale, timed up and go, timed stance test, six-minute walk test, steps in 360-degree turn, Movement Assessment Battery for Children (2^nd^ Edition), step test, ABILHAND assessment, active straight leg raise, and circumferential measures of the upper limb were reported as valid/reliable in limited populations and settings (many with small sample sizes). Participants appeared to embrace telehealth technology use, with most studies reporting high levels of participant satisfaction. If given a choice, many reported a preference for in-person physiotherapy assessments. Some inconsistencies in visual/auditory quality and challenges with verbal/non-verbal communication methods were reported. Telehealth was considered relatively cost-effective once services were established.

**Conclusions:**

Performing physiotherapy assessments using synchronous forms of telehealth appears valid and reliable for specific assessment types in limited populations. Further research is needed in all areas of physiotherapy practice, to strengthen the evidence surrounding its clinometric value. Clinicians contemplating using this assessment mode should consider the client/family preferences, assessment requirements, cultural needs, environment, cost considerations, access and confidence using technology.

**Protocol registration:**

PROSPERO: CRD42018108166.

Reduced or delayed access to health care services can have detrimental effects on the health and well-being of individuals [1]. Those living in regional, rural and remote areas with a lower population density are more likely to encounter reduced access to health care services [2], including access to physiotherapy services in both the public and private sectors [3,4]. This access limitation is particularly pronounced for more specialised areas of physiotherapy, such as those that address disability and paediatrics [5].

The use of telehealth services to improve physiotherapy access is increasing due to its ability to overcome the barriers of distance [6], service provider availability [7] and capacity to provide safe care to community members during periods of time when disease transmission is of concern. Telehealth has been defined by the World Health Organisation (WHO) as ‘*the use of telecommunications and virtual technology to deliver health care outside of traditional health care facilities’* [8]. The information and communication technologies (ICT’s) used in the delivery of telehealth services allow the transmission of health information such as voice, data, and still or video images over short and long distances, without the need for recipients of care or health professionals to be co-located [9]. Telehealth includes delivery of assessment and diagnosis, treatment, and preventative and curative aspects of health care [9], and can be provided using synchronous methods (real-time), or asynchronous methods (store and forward) [10].

Physiotherapy consultations performed using synchronous forms of telehealth have become even more common in recent times, due to the COVID-19 pandemic. Maintaining access to physiotherapy services during this time has been challenging for many health care providers [11,12] where area lockdowns and distancing requirements have interrupted and limited face-to-face service provision [11], initiating a shift in the way physiotherapy services are provided to clients [13-15]. Many health services and/or professional associations, such as the Australian Physiotherapy Association, NHS and Chartered Society of Physiotherapy (UK), have released advice and guidelines to support clinicians providing telehealth services during the COVID-19 pandemic [16,17]. Although this increased use of telehealth during the COVID-19 pandemic has been essential in ensuring continuity of care from many allied health and medical practitioners [14,18], concerns have been raised regarding the limited evidence supporting these changes to physiotherapy practice across the lifespan [13,15].

A physiotherapy client assessment, performed at commencement of physiotherapy service delivery and at regular intervals throughout the stages of intervention, provides vital information to therapists about the need for, and response to, therapeutic interventions [19]. Such assessment is essential for treatment plan development, clinical reasoning processes, intervention selection, and implementation of effective treatment strategies with clients [20]. Where a physiotherapist is not available to provide a face-to-face assessment, assessment via telehealth may need to be considered.

A variety of studies have investigated the use of telehealth to perform physiotherapy assessment and interventions with adult populations [21-23] however, performing accurate physiotherapy assessments using this method can be challenging [24]. Two systematic reviews, both limited to the field of musculoskeletal (MSK) physiotherapy and with a focus on the adult population, have been conducted [19,25] to investigate the validity and reliability of conducting physiotherapy assessment via telehealth. In 2017, Mani and colleagues investigated the use of internet-based physiotherapy assessments within the MSK field of physiotherapy, concluding that it is feasible to use telehealth systems to assess swelling, range of movement, pain, muscle strength, balance, gait and functional outcomes in adults referred to physiotherapy [19]. In 2018, Grona and colleagues investigated the validity and reliability of synchronous videoconference use in the assessment and management of adults presenting with MSK conditions [25]. Grona and colleagues concluded that synchronous video-based telehealth systems could be viable when assessing and managing adults with MSK conditions but may have poor reliability for assessments of the shoulder joint and elbow joint, and for nerve tests, scar assessment and lumbar posture assessment [25]. A small number of systematic reviews on telehealth have been conducted in other settings such as neurology [26,27] and aged care [28], with a focus on health and activity, using asynchronous monitoring systems or wearable devices.

To enhance physiotherapy assessment and care, it is important to understand the reliability, validity and utility of performing synchronous telehealth assessments throughout all areas of physiotherapy practice, across the lifespan. To our knowledge, there has not been a recent review of the literature investigating this topic. Therefore, the aim of this systematic review was to determine the current clinometric value (reliability, validity and broader utility) of physiotherapy assessments delivered using synchronous forms of telehealth, considering the full breadth of fields of physiotherapy practice. A second aim was to identify which of the validity, reliability and utility studies have been conducted in real-world rural or remote contexts and to provide a summary of their findings.

## METHODS

### Systematic review

This systematic review protocol was registered with PROSPERO (CRD42018108166).

### Search strategy

A comprehensive search of key literature databases was undertaken on 17th December 2020 to identify studies that addressed the aims of this review (**Table 1**). A hand search of reference lists of included studies was conducted following this to identify additional studies for inclusion. Databases were searched for articles published between January 2010 and 17th December 2020 inclusive. This date range was adopted to ensure inclusion of studies using contemporary technologies. The initial search was not restricted by language.

**Table 1:** Literature database search strategy

Databases	Search terms
PubMed/MEDLINE The Cochrane Library Embase EBSCO (Academic Search Complete, AHFS Consumer Medication Information, CINAHL, Computers & Applied Sciences Complete, Education Research Complete, ERIC, Health Business Elite, Health Source – Consumer edition, Health Source: Nursing/Academic Edition, MasterFILE Premier and SPORTDiscus with full text)	Physiotherapist; OR physical therapist; OR physiotherapy; OR physical therapy; OR rehabilitation AND Telehealth; OR telemedicine; OR telecare; OR e-health; OR telemental; OR telehealth care; OR telemonitoring; OR telerehabilitation; OR telepractice AND Assess*; OR evaluat*; OR analys*; OR measur*; OR reliability; OR validity; OR utility.

### Inclusion and exclusion criteria

The inclusion and exclusion criteria used for the selection of studies in this review are listed in **Table 2**. Authors of studies that did not specify the discipline of the health professional who conducted the telehealth assessment were contacted to ascertain this detail.

**Table 2:** Inclusion and exclusion criteria

Study characteristics	Inclusion criteria	Exclusion criteria
**Study types**	Validity studies, reliability studies and studies examining the broader utility of performing physiotherapy assessments conducted using synchronous forms of telehealth	Studies of interventions, or assessments using asynchronous forms of telehealth, and those that contained insufficient methodological detail of the telehealth assessment
**Participants**	Physiotherapists or physiotherapy students (under the supervision of a physiotherapist)	Studies that involved only health professionals other than physiotherapists performing or interpreting assessments conducted using synchronous forms of telehealth
**Contexts**	Studies from any field of physiotherapy and any setting of physiotherapy clinical practice.	

### Study selection

Screening and study selection commenced following completion of the search process. References of articles identified in the search were imported into EndNote (Clarivate, Philadelphia, PA, USA) [29] and duplicates removed. Titles and abstracts were screened, with articles that were clearly ineligible excluded. Full texts of remaining articles were sourced and reviewed by two independent reviewers (CZ and RP) to determine final eligibility based on the inclusion and exclusion criteria, with reasons for exclusion noted. Any discrepancies were resolved through discussion and consensus, with a third reviewer (WH) available to be consulted if consensus could not be reached. Studies deemed eligible for inclusion in this review were retained and analysed. The results of the search, screening and selection process were recorded in a PRISMA flow diagram (Preferred Reporting Items for Systematic Reviews) [30]. The PRISMA Checklist [30] was completed and can be found in Table S1 in the **Online Supplementary Document**.

### Quality assessment

The methodological quality of validity and reliability studies included in this review was assessed by two independent reviewers (CZ and VS) using the Brink and Louw critical appraisal tool (CAT) [31]. A third researcher (RP) was available to be consulted regarding any discrepancies that could not be resolved by discussion or consensus. The Brink and Louw CAT was developed in 2011 and was based on the Quality Appraisal of Diagnostic Reliability Studies (QAREL) and Quality Assessment of Diagnostic Accuracy Studies (QUADAS) tools [31,32]. The CAT was designed for use in systematic reviews that investigate validity and reliability of assessment tools. It uses a series of 13 questions scored as ‘yes’, ‘no’ or ‘not applicable’, with five of the items referring to validity and reliability, four items specific to validity and the remaining four items specific to reliability [31]. The CAT does not provide a quality rating [32]. To provide a quality rating for each study, we calculated a quality percentage and subsequent rating (**Table 3**) based on the number of items where ‘yes’ was given as the response, divided by the total number of items available to be scored, depending on the study type. Similar rating scales have been used by other authors when employing the Brink and Louw CAT, reporting a score of ≥60% as indicating ‘high quality’ [32-35].

**Table 3:** Quality ratings for methodological quality assessment

Quality rating (score range %)				
Very poor (0%-19%)	Poor (20%-39%)	Moderate (40%-59%)	Good (60%-79%)	Very good (≥80%)

The methodological quality of the utility studies included in this review was similarly assessed by two independent reviewers (CZ and VS), using the Mixed Methods Appraisal Tool (MMAT), version 2018, which is a critical appraisal tool developed by Hong and colleagues [36]. The MMAT is used to appraise the quality of various types of empirical studies [37]. The results are based on ratings for five criteria [37]. An overall rating was determined based on recommendations from Hong [38], who suggested a percentage rating for the number of ‘yes’ responses could be used if a quality rating score was required. These percentage scores were converted to quality descriptors, equivalent to those used for the Brink and Louw CAT (**Table 3**). The level of agreement between the two raters in ratings of methodological quality was ascertained by calculation of a Cohen’s kappa coefficient (k) [39].

Case studies or case series that were identified as eligible for inclusion in this review were analysed descriptively and did not undergo a separate methodological quality rating, being considered to provide low level (IV) evidence [40].

### Data extraction, synthesis and analysis

Key data and outcomes from included articles relating to the study design and analysis, aims, participants, area of physiotherapy practice and setting, assessment environment, assessment delivery and technology, assessment/outcome measures, and reliability, validity and utility of physiotherapy assessments conducted via synchronous telehealth, were extracted manually by a single reviewer. This data was summarised in a Microsoft Excel (Microsoft Inc, Seattle, WA, USA) spreadsheet to ensure a standard approach, to examine variation and for ease of analysis. Findings were synthesised using a critical narrative approach in which the strength of evidence from individual studies was considered, based on the methodological quality of each study. Findings of individual studies were reported in tables and figures with particular consideration given, in the reporting of results, to findings from studies performed in real-world rural or remote contexts rather than simulated contexts. A meta-analysis was not conducted due to the clinical and methodological diversity in the types and methods of telehealth assessments used and heterogeneity of the reported outcomes of the studies included in the review.

### Deviations from the protocol

Some minor deviations from the original protocol published with PROSPERO (CRD42018108166) were made to improve the rigor of this review. The date range for the original protocol was altered, with search originally to be conducted in August 2018. The search was subsequently completed in December 2020. The inclusion and exclusion criteria were further refined to focus on synchronous forms of telehealth. The MMAT was also included as the critical appraisal tool used to appraise utility studies included in this review. Finally, one further author (VS) who was integral in completing this review was added to the author list.

## RESULTS

**Figure 1:**
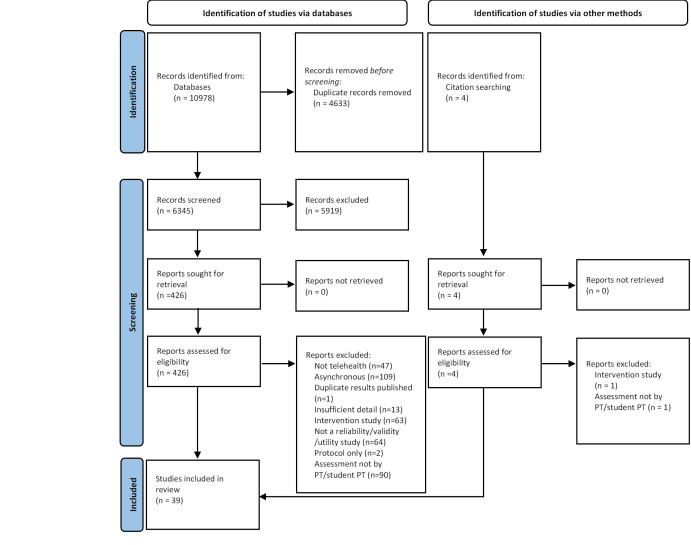
PRISMA flow diagram summarising results of the search, screening and selection processes [30].

The results of the search, screening and selection process can be found in **Figure 1**. Thirty-nine articles were retained following screening and selection. The third reviewer was not required for the small number of articles where there was initial disagreement regarding eligibility (n = 5), as consensus regarding eligibility was reached through discussion between the two primary reviewers.

### Study types and methodological quality

The methodological quality of each included study is summarised in **Table 4**. There was a strong level of agreement between the two reviewers’ scores, k = 0.90 (95% CI, 0.85 to 0.96), *P* < 0.001. Of the 39 articles reviewed, 18 studies investigated the concurrent validity and/or reliability of synchronous telehealth physiotherapy assessments (**Table 4**). The methodological quality of the validity and reliability studies ranged from ‘good’ (n = 10) to ’very good’ (n = 8). Most studies described participants in detail (n = 13) and the index test in enough detail to enable replication (n = 12). The statistical methods used within each article, although varied, were appropriate to the research question. One study failed to explain participant withdrawals and one did not provide qualification details of the raters involved.

**Table 4:** Summary of characteristics of included validity, reliability and utility studies

First author & year; Area of PT practice	Title	Study design	Relevant analysis used	Participants	Telehealth assessment environment	Telehealth assessment delivery & technology	Quality rating (%) and descriptor
Avelino, 2020 [41]; Neurological	Validation of the telephone-based application of the ABILHAND for assessment of manual ability after stroke.	Validity study: Cross-sectional design.	Descriptive, mean difference, Intraclass Correlation Coefficient (ICC), weighted Kappa (κ), 95% confidence interval (CI).	102 participants (49% male, mean age 65yrs ±13) with stroke (mean time 37 d post ±38) recruited from the community in Brazil.	Participants completed the ABILHAND assessment F2F and by telephone 5-7 d apart. Further details on location of participant not provided.	Synchronous telephone telehealth consultation. No further details described.	67%, Good
Boggs, 2020 [42]; Cancer Care	Telehealth and physical therapy clinical decision making in a patient with a falcine meningioma.	Utility study: Case report.	Descriptive.	A 50-y-old male presenting with 2-d history of left-sided numbness/tingling.	Further details of the location of participant not provided.	Synchronous telephone telehealth consultation.	Case report, N/A
Cabana, 2010 [43]; Musculo-skeletal	Interrater agreement between telerehabilitation and face-to-face clinical outcome measurements for total knee arthroplasty.	Interrater reliability study.	Krippendorff’s α reliability estimate, mean difference between methods (%) (95% CI).	15 participants (8 males, mean age 62 y) recently discharged after total knee arthroplasty surgery.	Home setting via telehealth (n = 9) or second clinical environment (n = 6). Research assistant was on-site for safety.	Synchronous VC telehealth consultation. 2x H264 VC Coder Decoders (Tandberg 500 MXP, Canada), integrated wide-angle cameras, 20-inch LCD TVs, modular software interface.	63%, Good
Cary, 2016 [10]; General	Benefits and challenges of delivering tele-rehabilitation services to rural veterans.	Utility study: Qualitative design.	Qualitative thematic analysis (2 therapist discussion groups).	6 clinicians (17% male, one PT) providing telehealth services to veterans in their homes (TeleHOME project, North Carolina).	In-home safety and mobility assessment by an occupational therapist/PT via telehealth with a LNP present.	Synchronous VC, asynchronous photos/video recordings. Encrypted laptop/ iPad (Apple Inc, Cupertino, CA, USA) internal/USB webcam, Cisco/Jabber (Movi) (Cisco Systems Inc, San Jose, CA, USA), WiFi/wireless services, video camera & tripod.	20%, Poor
Conlan, 2016 [44]; Women’s health	An exploration of the efficacy of telehealth in the assessment and management of stress urinary incontinence among women in rural locations.	Utility study: Case report.	Descriptive with use of medians and frequencies.	6 women (mean age 36.5yrs) with self-reported stress urinary incontinence from rural towns in Western Australia.	Home setting.	Synchronous VC and telephone consultations. PT used Microsoft LifeCam HD-3000 and Skype (Microsoft Inc, Seattle, WA, USA), and telephone. Participants used own device in their home.	Case report, N/A
Cottrell, 2018 [45]; Advanced practice	Agreement between telehealth and in-person assessment of patients with chronic musculoskeletal conditions presenting to an advanced-practice physiotherapy screening clinic.	Interrater agreement study: Repeated measures.	Descriptive statistics, diagnosis rated as same/similar/ different. Exact agreement, % specific agreement, Cohen’s κ and Gwet’s AC1.	42 participants with lumber spine (n = 14), shoulder (n = 14) or knee (n = 14) conditions from a Neurosurgical & Orthopaedic Physiotherapy Screening Clinic (Brisbane).	‘Simulated’ telehealth environment. F2F and telehealth assessments in separate rooms in the same hospital.	Synchronous VC. Dell (Dell, Round Rock, TX, USA) computer connected to hospital's network, e-HAB TR videoconferencing (NeoRehab, Brisbane, Australia), participants used iPad (Apple Inc, Cupertino, CA, USA), stand and internet connection.	88%, Very good
Cottrell, 2021 (First published July 2019) [46]; Advanced practice	Comparing fly-in fly-out and telehealth models for delivering advanced-practice physiotherapy services in regional Queensland: An audit of outcomes and costs.	Utility study: Retrospective electronic medical record audit.	Descriptive statistics and cost comparison (average cost per slot), 95% CI, weighted average ratios and estimated net financial position.	26 telehealth participants (62% female, mean age 53.8 ± 11.4yrs) with spinal injury. 18 F2F participants (83% female, mean age 46.6 ± 13.8yrs) with upper/ lower limb (72.2%) or spinal (27.8%) concerns.	Telehealth assessment conducted in a regional facility with a graduate PT in attendance and clinical leader (PT with advanced training) located remotely in a metropolitan tertiary hospital.	Limited details provided on the telehealth consultation including how assessment was conducted. Computer, webcam and Movi software (Cisco Systems Inc, San Jose, CA, USA) appear to have been used.	80%, Very good
Cox, 2013 [47]; Cardio-respiratory	Assessing exercise capacity using telehealth: a feasibility study in adults with cystic fibrosis.	Feasibility and repeatability study.	Paired *t* test (significance set to *P*<.05) and Bland and Altman mean (±SD).	10 adults with cystic fibrosis (50% male, mean age 32 ± 7yrs), mean FEV_1_ = 55.4%.	‘Simulated’ telehealth environment. F2F and telehealth assessments in separate rooms of the same building.	Synchronous VC. Desktop computer, webcam, video-collaboration software (VSee, Sunnyvale, CA, USA), pulse oximeter, participants received pictorial and written instructions.	75%, Good
Demmelmaier, 2010 [48]; Musculo-skeletal	Physiotherapists' telephone consultations regarding back pain: a method to analyse screening of risk factors.	Utility study: An exploratory study investigating PT telehealth assessments and the reliability of a research protocol.	Descriptive statistics and means, ICC and κ values calculated pairwise to estimate agreement.	5 PT’s (100% female, 25-55yrs, 3-31yrs clinical experience). 17 consultations were reviewed.	Telephone consultations with patients calling a physiotherapy clinic in two urban communities in Sweden. No location details provided.	Synchronous telephone telehealth consultation recorded using a Grundig Stenocassett Recorder (Dt 3400) (Grundig, Neu-Isenberg, Germany) or a ZAP Digital Voice Recorder-X1 and were transcribed verbatim.	40%, moderate
Eannucci, 2020 [49]; General	Patient satisfaction for telehealth physical therapy services was comparable to that of in-person services during the COVID-19 pandemic.	Utility study: Retrospective review of survey data.	Means, standard deviations, frequencies and % used with comparisons using one-way ANOVA and Chai squared. Kruskal-Wallis tests with significance = 0.05.	1147 participants (67% female, mean age 60.3 ± 15yrs) receiving either F2F or telehealth services at the Hospital for Special Surgery. 133 initial and 104 follow-up physiotherapy telehealth consultations.	Telehealth assessments occurring following shelter in place orders due to COVID-19. No further details regarding the telehealth assessment environment were provided.	No details regarding the telehealth assessment delivery or technology used were provided.	60%, good
Exum, 2020 [50]; Cardio-respiratory	Applying telehealth technologies and strategies to provide acute care consultation and treatment of patients with confirmed or possible COVID-19.	Utility study: Case report of the health care (including PT) response to COVID-19 in an acute care setting.	Descriptive analysis.	11 PT’s recruited in the COVID-19 response team involved in triage, developing telehealth assessment and intervention strategies where possible.	Consultations performed remotely or outside patient’s room with therapist viewing consultation through a window where required. A registered nurse was with the patient when required.	Synchronous VC or telephone consultations using Apple iPad/iPad Air 2 or 6th Generation iPad (Apple Inc, Cupertino, CA, USA), Microsoft Teams (Microsoft Inc, Seattle, WA, USA) and hospital provided laptop (Health Insurance Portability and Accountability Act compliant).	N/A, case report
Funderskov, 2019 [51]; Palliative care	Telemedicine in specialised palliative care: Healthcare professionals’ and their perspectives in video consultations – A qualitative study.	Utility study: Explorative qualitative study of health professionals’ experiences.	Malterud’s systematic text condensation with thematic coding and categorisation.	8 health care professionals (one PT) were included in the study who had conducted 82 telehealth consultations for patients with severe illness.	In-home consultations with community nurses in attendance and therapists attending remotely via telehealth.	Synchronous VC using tablets provided to participants with Sim cards (able to accept 4G), desktop computer and developed App for relatives to participate when unable to be with the participant in person.	80%, very good
Galiano-Castillo, 2014 [52]; Cancer care	Agreement between telerehabilitation involving caregivers and face-to-face clinical assessment of lymphedema in breast cancer survivors.	Criterion validity and inter-rater reliability study.	Bland and Altman limits of agreement, mean difference, Cronbach's α and ICC’s (Rho). Inter-rater reliability determined by two-way random effect ICC coefficients (Rho) and CI’s.	30 female breast cancer survivors (mean age 46.33 ± 9.05yrs, 66.7% lumpectomy, 26.7% unilateral mastectomy, 6.7% bilateral mastectomy). 80% unilaterally and 20% bilaterally affected.	‘Simulated’ telehealth environment. Assessments were conducted on the same day within a 120-min interval at the University of Granada (Spain), with a caregiver completing the telehealth assessment.	Synchronous VC with e-CUIDATE web-based system, internet connection (bandwidth 256 Kbit/s), two personal computers and Wormhole Web Conference (Wormhole IT, San Jose, CA, USA) and Skype software (Microsoft Inc, Seattle, WA, USA).	100%, very good
Grona, 2017 [53]; Musculo-skeletal	Case report: using a remote presence robot to improve access to physical therapy for people with chronic back disorders in an underserved community.	Utility study: Case report.	Descriptive using thematic analysis.	45-y-old, female with a chronic back disorder (20yr history of low back pain, post L5-S1 microdiscectomy & laminectomy).	LNP present during the assessment (same room as participant). PT joined via the remote presence robot.	Synchronous VC via a remote presence robot (RP-7) (InTouch Health, Santa Barbara, CA, USA) which contained a screen (+ screen sharing), 2 × cameras and microphone. LNP was present. No further details provided.	N/A, case report
Harland, 2017 [54]; Musculo-skeletal	Physiotherapists and general practitioners’ attitudes towards 'PhysioDirect' phone based musculoskeletal physiotherapy services: a national survey.	Utility study: Questionnaire based exploratory study capturing databased on a randomised controlled trial.	Percentage response rates.	541 PT (82% female, 23% working within and a phone-based service) and 68 general practitioner respondents who refer to these services (68% female).	Telephone consultations with patients calling the PhysioDirect service from the United Kingdom (UK).	Synchronous telephone physiotherapy service. No further details of technology provided.	100%, very good
Hollinghurst, 2013 [55]; Musculo-skeletal	A pragmatic randomised controlled trial of 'PhysioDirect' telephone assessment and advice services for patients with musculoskeletal problems: economic evaluation.	Utility study: Economic evaluation and cost-utility analysis based on a randomised controlled trial.	Frequencies, means, medians, Quality Adjusted Life Years & cost-effectiveness ratios (ICER), cost-effectiveness acceptability curve (CEAC) and net monetary benefit (NMB) estimated.	1506 PhysioDirect and 743 usual care participants. This study used complete cost and QALY data for 840 PhysioDirect and 432 usual care participants.	Telephone consultations occurred with participants (from Bristol, Somerset, Stoke-on-Trent and Cheshire) calling from their own home/community. Participants could call-back the service if required or wanted a F2F appointment.	Synchronous telephone assessment/triage system which used computerised assessment templates at times. No further details of technology provided.	20%, poor
Hwang, 2016 [56]; Cardio-respiratory	Assessing functional exercise capacity using telehealth: is it valid and reliable in patients with chronic heart failure?	Nested validity, inter- and intra-rater reliability study: Part of a randomised controlled trial investigating telehealth for heart failure.	Mean difference, paired *t* tests, ICC and Bland and Altman limits of agreement. Inter- and intra-rater reliability of telehealth assessments were investigated using ICC.	17 participants with stable chronic heart failure (88% male, mean age 69 ± 12yrs) with ischemic cardiomyopathy (65%), idiopathic cardiomyopathy (18%), heart failure (18%).	‘Simulated’ telehealth environment. Assessments occurred in a hospital setting (Brisbane, Australia) on the same day with examiner in a separate room during the telehealth assessment.	Synchronous VC (recorded for reliability) using laptop (Dell Inspiron 15), VC software (Adobe Connect 9.2) (Adobe Inc, San Jose, CA, USA), internet connection (3G wireless broadband). Participants received pictorial and written instructions on using the technology provided.	69%, good
Kinder, 2019 [57]; Women’s health	Telerehabilitation for treating pelvic floor dysfunction: a case series of 3 patients' experiences.	Utility study: Case series with one participant completing a physiotherapy assessment via telehealth.	Descriptive analysis.	One female participant (41yrs, 4-mo postpartum) experiencing stress urinary incontinence, diastasis recti and needing pelvic floor muscle training.	Conducted in a suburb/city. No further details provided.	Synchronous VC using BlueJay Engage App (BlueJay Mobile Health, Livermore, CA, USA) which required setup and routine updates. Participant used their own iPhone device (Apple Inc, Cupertino, CA, USA).	N/A, Case series
Lade, 2012 [58]; Musculo-skeletal	Validity and reliability of the assessment and diagnosis of musculoskeletal elbow disorders using telerehabilitation.	Validity and reliability study: Repeated measures.	Descriptive analysis with percentage of exact or similar agreement, χ^2^ and weighted κ.	10 participants (9 males, mean age 38 ± 13yrs), 11 elbow cases (1 × bilateral) recruited from a private musculoskeletal and Sports Injury Clinic (Queensland, Australia).	‘Simulated’ telehealth environment. Participants were assessed in a single session, F2F and via telehealth (examiner in a separate room).	Synchronous VC (with recording capabilities) using telerehabilitation system (eHAB, NeoRehab, Brisbane, Australia) allowing videoconferencing (320x240 pixels) and Wireless 3G internet connection.	69%, good
Lovo, 2019 [59]; Musculo-skeletal	Experience of patients and practitioners with a team and technology approach to chronic back disorder management.	Utility study: Qualitative design based on a randomised controlled trial pilot study.	Proportions, medians and interquartile ranges (IQR) of participants responses to a survey.	19 participants (mean age 50.84 ± 13.87, 57.9% female) with low back ± leg pain. Six participants and two health practitioners (LNP and PT) participated in a semi-structured phone interview.	A lumbar spine physiotherapy assessment was completed by an urban PT via telehealth. A LNP was in attendance with the participant.	Synchronous VC using a laptop with VidyoDesktop Software Inc. (USA) and external camera (pan, tilt & zoom capabilities) at LNP and participant end. No further details provided.	100%, very good
Mani, 2019 [60]; Musculo-skeletal	Concurrent validity and reliability of telerehabilitation-based physiotherapy assessment of cervical spine in adults with non-specific neck pain.	Concurrent validity, intra-rater and inter-rater reliability study: Repeated measures.	Bland-Altman’s limit of agreement, standard error of measurement (SEM), coefficient of variation, minimal detectable changes, ICC, % agreement and χ^2^ stats.	11 (8 females, mean age 32.7 ± 10.9yrs) with non-specific neck pain. Nine participant recordings were used for reliability analysis.	‘Simulated’ telehealth environment (physiotherapy clinic, University Kebangsaan Malaysia). Participants accompanied by relative/friend or clinical assistant were assessed in a single session via F2F and telehealth (examiner in a separate location).	Synchronous VC using TelePTsys system allowing videoconferencing (320x240 pixels) with e-Goniometer, e-Ruler and scheduling and reporting software. Digital image capture ability, MPEG-4 video and Speex audio codecs (Xiph.Org Foundation, Somerville, MA, USA) and 15.5-inch laptop with Logitech webcam (C310) (Logitech, Fremont, CA, USA), with inbuilt microphone with noise reduction (patient-end).	77%, good
Mehta, 2020 (First published in October 2020) [61]; Musculo-skeletal	Virtual assessments of knee and wrist joint range motion have comparable reliability with face-to-face assessments.	Cross-sectional reliability study.	Paired *t* tests (*P* < 0.05 significance), ICC with 2-way random effects model, SEM and Bland-Altman levels of agreement and mean difference.	54 PT students and known acquaintances (30 females, mean age 24.5 ± 1.9 y) without musculoskeletal impairments of upper or lower limbs.	‘Simulated’ telehealth environment. Assessment occurred in a single session in a University setting (USA). Virtual assessment first, followed by a F2F.	Synchronous VC consultation using wall-mounted camera system, Zoom Cloud Meetings ® (Zoom Video Communications, Inc., San Jose, CA, USA), 12-inch and 6-inch 360 degrees universal goniometer (F2F only). Range of movement was visually estimated in the telehealth group.	78%, good
Mukaino, 2020 [62]; Cardio-respiratory	An affordable, user-friendly telerehabilitation system assembled using existing technologies for individuals isolated with COVID-19: Development and feasibility study.	Utility study: Feasibility using a convenience sample with small telehealth assessment component.	Descriptive analysis using means and standard deviations.	10 participants (60% female, mean age 60 ± 18yrs) admitted to a University Hospital diagnosed with COVID-19.	Telehealth assessment occurred in private isolated hospital room.	Synchronous VC consultation using desktop and tablet computer, pulse oximeter (Ring 2, Neuroceutcal Inc), Zoom (Zoom Video Communications, Inc., San Jose, CA, USA), Skype (Microsoft Inc, Seattle, WA, USA) and Team Viewer (TeamViewer GmbH, Germany).	40%, moderate
Nicola, 2018 [63]; Paediatrics	The feasibility and concurrent validity of performing the Movement Assessment Battery for Children - 2nd Edition via telerehabilitation technology.	Concurrent validity study using a test-retest method to compare telehealth with a F2F assessment.	Agreement determined by mean absolute difference (MAD), % agreement and Bland-Altman limits of agreement.	59 typically developing children (5-11 y, 28 females) attending a local public school (Queensland, Australia).	Telehealth assessment conducted in a school setting with PT located off site (teacher aid was present at school). Both assessments occurred sequentially in one day when possible.	Synchronous VC using the eHAB telerehabilitation system (NeoRehab, Brisbane, Australia) accessed at the school campus via an iPad (Apple Inc, Cupertino, CA, USA) and a portable 4G Wi-Fi hotspot.	100%, very good
O’Donovan, 2020 [64]; Musculo-skeletal	Telehealth for delivery of haemophilia comprehensive care during the COVID-19 pandemic.	Utility study: Narrative design of a multi-disciplinary haemophilia service.	Descriptive analysis.	38 patients with severe haemophilia who obtain services from the European Haemophilia Comprehensive Care Centre responded to a survey about telehealth services.	Telehealth assessments occurred remotely with 49% located in Dublin and 51% outside of Dublin. No further details provided.	Telephone and VC using Blue Eye (RedZinc Service, Ireland) video communication system.	N/A, narrative study
Palacin-Marin, 2013 [65]; Musculo-skeletal	Agreement between telerehabilitation and face-to-face clinical outcome assessments for low back pain in primary care.	Validity, inter- and intra-rater reliability study: Repeated measures crossover design.	Bland-Altman limits of agreement, mean difference, Cronbach α and ICC.	15 participants (6 males, mean age 37yrs).	Participants attended a primary care centre where telehealth and F2F assessments were conducted using a set protocol.	Synchronous VC (recorded for reliability) using TPLUFIB-WEB web-based system, Skype software (Microsoft Inc, Seattle, WA, USA), Logitech HD Pro webcam C920 (Logitech, Fremont, CA, USA), Kinovea software and computers.	85%, very good
Pearson, 2016 [66]; Musculo-skeletal	Acceptability to patients of PhysioDirect telephone advice and treatment services: a qualitative investigation.	Utility study: Nested qualitative, within a randomised controlled trial.	Thematic, cross-sectional analysis.	82 participants (46% male, mean age 58yrs ±16.88) from the PhysioDirect randomised controlled trial were interviewed once (most at home), following an episode of service.	Telephone consultations with participants (from Bristol, Somerset, Stoke-on-Trent and Cheshire) located in their home/community. Service users could call-back the service for support or a F2F appointment.	Synchronous telephone assessment/triage system which used computerised assessment templates at times. No further details of technology provided.	60%, good
Peterson, 2018 [67]; Musculo-skeletal	Use of a modified treatment-based classification system for subgrouping patients with low back pain: agreement between telerehabilitation and face-to-face assessments.	Repeated measures agreement study.	Overall and frequency of percent agreement, κ coefficient and 95% CI. Wilcoxon signed-rank test examined significant difference, power, and effect sizes.	47 participants (mean age 48.6 ± 15yrs, 30% male) with <90-d duration of low back pain symptoms recruited from two outpatient private practice clinics in Arizona.	‘Simulated’ telehealth environment. All assessments completed in a private practice, 10-min apart within the same room.	Synchronous VC using web-based teleconference application Zoom (Zoom Video Communications, Inc., San Jose, CA, USA), two personal computers and an iPad (Apple Inc, Cupertino, CA, USA) mounted with wide-angle lens and attached to a tripod.	83%, very good
Prada, 2020 [68]; Neurological	The suspected SARS-Cov-2 infection in a Charcot-Marie-Tooth patient undergoing postsurgical rehabilitation: the value of telerehabilitation for evaluation and continuing treatment.	Utility study: Case report.	Descriptive analysis.	One participant (28yrs, male) with Charcot-Marie-Tooth post tendon transfer surgery transitioned to telehealth services after contracting COVID-19.	Assessment occurred remotely after showing signs of COVID-19. No further specific details on location provided.	Synchronous VC using publicly available software. No further details provided.	N/A, case report
Richardson, 2017 [69]; Musculo-skeletal	Physiotherapy assessment and diagnosis of musculoskeletal disorders of the knee via telerehabilitation.	A criterion validity, inter-and intra-rater design: repeated measures.	Percentage exact agreement, percentage similar agreement, χ^2^ or weighted κ and descriptive analysis.	18 participants (8 males, mean age 23 ± 7yrs) with knee pain presenting to a private practice (Brisbane, Australia).	‘Simulated’ telehealth environment. Assessed in one session using F2F and telehealth methods (10min break between). Assessment items chosen by examiners. Telehealth assessment was carried out by a remote PT (location unspecified).	Synchronous VC (recorded for reliability) using eHAB TR system (Version 2; NeoRehab, Brisbane, Australia), Wi-Fi broadband connection on 3G mobile network and pre-recorded video and still images used for demonstration purposes.	62%, good
Russell (a), 2010 [4]; Musculo-skeletal	The diagnostic accuracy of telerehabilitation for non-articular lower-limb musculoskeletal disorders.	A validity, inter-rater and intra-rater design: repeated measures.	χ^2^ or weighted κ and percentage of exact agreement or percentage of similar agreement and descriptive statistics.	19 participants (5 males, mean age 26 ± 13yrs, from a university student population) presenting to a private practice (Brisbane, Australia) with lower limb pain.	‘Simulated’ telehealth environment. Assessed in one session with F2F and telehealth assessment methods (location unspecified). Assessment items chosen by examiners.	Synchronous VC (recorded for reliability) using eHAB TR system (Uniquest, Brisbane, Australia), remote camera pan and zoom features and 3G Internet connections (Telstra Next G; Melbourne, Australia).	85%, very good
Russell, 2013 [70]; Neurological	Internet-based physical assessment of people with Parkinson disease is accurate and reliable: a pilot study.	Validity, inter-rater and intra-rater reliability study: simultaneous assessment.	Bland and Altman limits of agreement and MAD, weighted κ scores, ICC’s, percentage exact agreement and agreement within one point (%A±1).	12 participants (50% male, mean age 66.1 ± 8.5yrs) with Parkinson disease (rated I-IV on Hoehn & Yahr Score).	‘Simulated’ telehealth environment. Participants assessed via telehealth and F2F simultaneously (location unspecified). Two therapists were in the same room as the participant (remote therapist was located in an alternate room).	Synchronous VC (recorded for reliability) using eHAB TR system (Uniquest, Brisbane, Australia), and 3G Internet connections (Telstra Next G; Melbourne, Australia).	69%, good
Russell (b), 2010 [71]; Musculo-skeletal	Telerehabilitation mediated physiotherapy assessment of ankle disorders.	Validity, inter-rater and intra-rater design: repeated measures.	Percentage agreement, weighted κ, Percentage exact agreement, percentage similar agreement and χ^2^.	15 participants (5 males, mean age 24.5 ± 10.8yrs) who presented to a musculoskeletal clinic (Brisbane, Australia) with ankle pain were included.	‘Simulated’ telehealth environment. Participants attended a single session. F2F and remote assessments completed with 10min break between methods. Assessment items chosen by examiners.	Synchronous VC (recorded for reliability) using eHAB TR system (Uniquest, Brisbane, Australia), 3G Internet connections (Telstra Next G; Melbourne, Australia) with pre-recorded videos of tasks shown to participants as required ie, ligament stability tests.	77%, good
Salisbury, 2013 [72]; Musculo-skeletal	Effectiveness of PhysioDirect telephone assessment and advice services for patients with musculoskeletal problems: pragmatic randomised controlled trial.	Utility study: Based on a Randomised controlled trial comparing PhysioDirect to usual care.	Descriptive statistics and multivariable regression models.	1506 participants (PhysioDirect) and 743 (usual care) (40% male, mean age 60yrs) with 30% lower limb, 27% lumbar spine, 14% thoracic or cervical spine and 23% upper limb concerns.	Participants in the PhysioDirect group were invited to contact PT’s by telephone within specified days/times to access the service from home, place of work or other area within the community.	Synchronous telephone assessment/triage system using computerised assessment templates at times. Following the initial telephone consultations, participants could call-back the service if they had further problems or wanted to request a F2F appointment.	80%, very good
Steele, 2012 [73]; Musculo-skeletal	Assessment and diagnosis of musculoskeletal shoulder disorders over the internet.	Validity, inter-rater and intra-rater reliability design: repeated measures.	Percentage agreement (same, similar and different) and percentage exact and close agreement χ^2^ and weighted κ.	22 participants and 28 assessments (16 males, mean age 30.7 ± 14.2yrs, some with bilateral shoulder pain). Participants (students and staff) were recruited from the University Musculoskeletal Clinic.	‘Simulated’ telehealth environment. Participants underwent a F2F and telehealth assessment in one session (Queensland, Australia). Examiner was in an alternate room and chose the assessment items.	Synchronous VC (recorded for reliability) using the eHAB TR system (Neorehab, Brisbane, Australia) and 3G Internet connections (Telstra Next G; Melbourne, Australia).	92%, very good
Truter, 2014 [74]; Musculo-skeletal	The validity of physical therapy assessment of low back pain via telerehabilitation in a clinical setting.	Validity study: repeated measures.	χ^2^ tests, percentage exact agreement, Pearson correlation (r), Paired *t* test and κ (level of significance, *P*<0.05).	26 participants (11 males, mean age 43yrs) presenting with low back pain from a regional town (Queensland, Australia).	‘Simulated’ telehealth environment. Participants attended telehealth and F2F assessments in one session (10-15mins apart) at a rural hospital. A friend/ allocated untrained helper assisted.	Synchronous VC using eHAB system (version 2, Neorehab, Brisbane, Australia) with recording capability, store and forward images, remote camera pan, zoom and 3G Internet connections (Telstra Next G; Melbourne, Australia).	100%, very good
Turner, 2018 [75]; Musculo-skeletal	The validity of physical therapy assessment of low back pain via telerehabilitation in a clinical setting.	Utility study: Case series design.	Descriptive analysis.	3 participants (45yr old male with LBP, a 49yr old female with right sided upper cervical pain and a 50yr old male with right lateral elbow pain).	Rural hospital setting. Elements of the assessment were completed asynchronously prior to the telehealth appointment.	Synchronous VC. Using participants own smartphone or tablet devices with internet connection, encrypted HIPAA compliant application (not specified) and a laptop with secure internet connection (clinician).	100%, very good
Wood, 2017 [76]; Cardio-respiratory	Telehealth clinics increase access to care for adults with cystic fibrosis living in rural and remote Western Australia.	Utility study: Single group.	Descriptive, paired *t* tests, Wilcoxon signed rank tests used. Positive or negative binomial regression compared utilisation data.	23 adults with cystic fibrosis (61% female, mean age 31.4 ± 10.2yrs) living in rural or remote areas of Western Australia. 17 participants responded to the survey.	Telehealth clinic located at the participants nearest regional hospital with clinic staff calling in remotely from Perth (Australia).	Synchronous VC using Polycom HDX ® series (Polycom, San Jose, USA).	100%, very good
Wootton, 2020 [77]; Cardio-respiratory	COVID-19 rehabilitation delivered via a telehealth pulmonary rehabilitation model: a case series.	Utility study: Case series.	Descriptive analysis.	3 participants (100% male, ages 59-80) who tested positive for COVID-19.	Assessments completed in the home setting with therapist in an alternate location.	Synchronous VC used to administer the exercise test and review intervention. Questionnaires were completed asynchronously. Participants used their own device. Oxygen saturation and heart rate monitors were sent to their home prior to the assessment.	N/A, case series

F2F – face-to-face, LNP – licensed nurse practitioner, VC – videoconference, N/A – not appraised, PT – physiotherapist, TR – telerehabilitation

Among the studies that investigated the concurrent validity of conducting a telehealth assessment when compared to a face-to-face assessment (n = 15), some failed to explain the face-to-face assessment (n = 3) or it’s execution in enough detail to allow replication (n = 6). Most studies (n = 13) included a face-to-face assessment that was completed independent of the telehealth assessment and all studies considered a time frame between the two assessments that was short enough to limit changes in assessment findings.

Eleven of the included studies investigated the inter-rater and/or intra-rater reliability of performing a physiotherapy assessment via telehealth. Most study raters were blinded to the results of other raters (n = 10). Only one of the nine intra-rater reliability studies mentioned that raters were blinded to their own findings. The order of the examinations was varied in nine of the eleven studies. Three studies investigated inter-rater agreement between a clinician conducting a physiotherapy assessment via telehealth and a clinician conducting the same assessment face-to-face [43,45,67]. These studies demonstrated either good (n = 1) or very good (n = 2) methodological quality. One study did not discuss the reference standard in detail, and another failed to blind raters to each other’s findings or set an appropriate time interval for test replication. Further details of the quality appraisals for the validity and reliability studies included in this review can be found in Table S2 in the **Online Supplementary Document**).

Twenty-one of the reviewed studies were broader utility studies, which reported on the feasibility and outcomes of conducting telehealth assessments across a range of physiotherapy practice areas. Twelve of the utility studies were appraised using the MMAT [36] and nine were case studies or descriptive papers which were not appraised (**Table 4**). Similarly, one further utility study [64] included in this review was not appraised as it did not meet the screening criteria for the MMAT. The studies that were not appraised were considered to provide only low level (IV) evidence.

The methodological quality of the utility studies that were appraised varied from ‘poor’ (n = 2) to ‘very good’ (n = 6) (Table 4). Five studies used a qualitative design which appeared to be appropriate for the study question. Some qualitative studies provided limited information, making it difficult to determine whether the findings were adequately derived (n = 2) or if results were authenticated from the data (n = 3). Further details of the results of the MMAT quality appraisals for the utility studies included in this review can be found in Table S3 in the **Online Supplementary Document**).

### Study characteristics

A summary of the characteristics and findings of the included studies is provided in **Table 4** and **Table 5**. These studies investigated the validity, reliability or broader utility of synchronous physiotherapy assessments performed via telehealth across a variety of clinical practice settings and populations, which included children and adult participants with and without motor impairments. Sixty-four percent of the studies were published over the past five years. The number of participants in each included study varied, with nine validity/reliability studies including less than 20 participants. Two larger studies investigated synchronous, telehealth physiotherapy assessments conducted in healthy populations [61,63]. Variability was also observed across the utility studies, with participant numbers ranging from one (case studies) to 2249 [72]. There was inconsistency in the execution of telehealth assessments across practice settings, with some participants accompanied by a friend or relative [50,60,74], teacher’s aide [63] or health practitioner (nurse or less experienced physiotherapist) [10,46,50,53,76] to assist with the assessment. The experience levels of clinicians conducting telehealth assessments also varied. Physiotherapy students under supervision performed telehealth assessments in some studies (n = 6), whilst others involved physiotherapists with extensive experience (up to 31 years) or post graduate training in specialised areas of practice [45,46]. Some clinicians had previous experience in performing virtual assessments or were provided with training prior to conducting the telehealth assessment, while others had none.

**Table 5:** Relevant aims and findings of included validity, reliability and utility studies

First author & year; area of PT practice	Study aims relevant to review	Relevant reported assessment / outcome measures	Key findings
Avelino, 2020 [41]; Neurological	Investigate concurrent validity of the ABILHAND telehealth assessment compared with a F2F assessment for individuals after stroke.	***Concurrent validity of:***	***Concurrent validity***
		ABILHAND assessment.	ABILHAND assessment: No significant difference observed between mean scores for telehealth and face-to-face assessments. Very high agreement for total scores (ICC = 0.90, 95%CI 0.85-0.93). Moderate agreement for most individual scores. Slight agreement with threading a needle and fastening a snap (κ≤0.20). Substantial agreement for washing hands, filing nails and buttoning trousers (κ = 0.61-0.66).
Boggs, 2020 [42]; Cancer Care	Describe the use of telehealth for triage, screening and clinical reasoning.	***Utility of telehealth:***	***Utility of telehealth***
		Subjective assessment and outcome.	Patient’s responses to telehealth subjective examination revealed a variety of red flags requiring further investigation. Referral was made to a medical practitioner based on red flags identified. Patient was subsequently diagnosed with right parietal parafalcine meningioma which was resected with no evidence of recurrence at 12-mo follow up.
Cabana, 2010 [43]; Musculo-skeletal	Explore reliability of outcome measures that can be used via telehealth in the home or clinical setting post total knee arthroplasty.	***Interrater reliability of:***	***Interrater reliability***
			Overall good interrater reliability for most measures except for scar condition.
		1. Knee range of motion (flexion and extension).	1. Knee range of motion: α = 0.80-0.85, MD = -6%.
		2. Scar condition.	2. Scar condition: α = 0.34, MD = -15%.
		3. Knee joint swelling (circumferential).	3. Knee joint swelling: α = 0.87, MD = 0%.
		4. 30s chair-to-stand test (strength).	4. Strength: α = 0.85, MD = 5%.
		5. Timed up & go.	5. Timed up & go: α = 0.86, MD = -6%.
		6. Gait (Tinetti Test) and Balance (Berg Balance Scale).	6. Gait: α = 0.79, MD = -2.5%. Balance: α = 0.76, MD = 1.7%.
Cary, 2016 [10]; General	Describe the TeleHOME program and identify clinicians’ perspectives of benefits & challenges to using this technology when providing therapy services to veterans residing in a rural area.	***Clinicians’ perspectives of utility of telehealth:***	***Clinicians’ perspectives of utility of telehealth***
		1. Technology and the interdisciplinary care processes.	1. Technology: Enabled collaboration.
		2. Challenges/ benefits encountered when working with other disciplines to deliver TeleHOME services.	2. Challenges/benefits of working with other disciplines: Onsite LNP allowed identification of unexpected issues, valuable upskilling, enhanced service provision, trans-disciplinary and collaborative practice.
		3. Interdisciplinary care processes.	3. Care processes: Opportunities for collaboration and efficiencies, standard equipment unavailable in the home setting, time consuming consent procedures, communication streamlined but not as efficient as home health services and documentation delayed. Communication strategies included turn taking, checking in and using mutually understood terminology.
		4. Overall benefits.	4. Overall benefits: Increased access for those living in underserved rural areas, enhanced coordination of care, communication and technology enhanced treatment delivery.
		5. Overall challenges.	5. Overall challenges: Unfamiliarity with technology and equipment, challenges in viewing, mobility assessments, connectivity issues or underpowered devices resulting in poor video quality or loss of communication, size of data files, limited access to specialised equipment, safety concerns and small teams covering large geographical region causing delays.
Conlan, 2016 [44]; Women’s health	Investigate satisfaction of physiotherapy telehealth consultations for women with stress urinary incontinence in rural areas and understand service access barriers.	***Utility of telehealth:***	***Utility of telehealth***
			Telehealth continence assessment may be effective in screening participants to determine if treatment via telehealth or direct contact would be efficacious.
		1. Satisfaction with telehealth assessment (anonymous survey with Likert response).	1. Satisfaction: 83% of participants ‘agreed’/ ‘strongly agreed’ with “I was satisfied with the telehealth interview process”.
		2. Barriers to accessing continence physiotherapists.	2. Barriers to service access: Inconvenience (median 2, IQR 2), site related factors (median 1.5, IQR 3), fear (median 1, IQR 3) and cost (median 1, IQR 1).
Cottrell, 2018 [43]; Advanced practice	Determine the levels of agreement for clinical management decisions, diagnosis and further investigations comparing telehealth and F2F assessment methods in an advanced practice physiotherapy screening clinic.	***Interrater reliability of:***	***Interrater reliability***
		1. Clinical management decisions and primary clinical diagnosis.	1. Clinical management decision: High levels of agreement for shoulder, knee and lumbar spine assessments (71.4%-92.9%, AC1 = 0.70-0.93). Referral to allied health professions agreement levels ranged from good to very high (76%-93% exact agreement, AC1 = 0.58-0.90). Primary clinical diagnosis reported as: same = 38.1%, similar = 45.2%, different = 16.7%.
		2. Further radiological or pathology investigations.	2. Decisions for further investigations: High levels of agreement with pathology (97.6%, AC1 = 0.97) and slightly lower levels for radiological (81%, AC1 = 0.74).
		***Satisfaction rating (VAS):***	***Satisfaction:***
		Confidence in using telehealth, would they recommend to a friend, is it as good as in-person assessment, visual clarity, audio clarity and overall satisfaction measured.	Overall high levels of satisfaction (mean = 89/100 VAS). Mean scores of >80 (mm) for individual questions except when asking if telehealth was as good as an in-person assessment (mean = 72/100 mm).
Cottrell, 2021 (First published July 2019) [46]; Advanced practice	Describe and compare the economic and service characteristics of a fly-in, fly-out model of care compared with telehealth in a Neurosurgical & Orthopaedic Physiotherapy Screening Clinic and Multidisciplinary Service.	***Utility of telehealth:***	***Utility of telehealth***
		1. Service metrics.	1. 33 appointment slots completed for each service type with similar utilisation. All consultations by the same clinical leader.
		2. Clinical actions.	2. Based on assessment results, some participants were referred for further radiology/pathology investigation (telehealth 25%, fly-in, fly-out 25%), medical practitioner review (telehealth = 25%, fly-in, fly-out = 8.3%) or referred to allied health (telehealth = 14, fly-in, fly-out = 19). Most remained with the Screening Clinic and Multidisciplinary Service (telehealth 66.7%, fly-in, fly-out 87.5%). Equal numbers of participants from each group had no specific treatment plan (n = 2), were discharged from the Neurosurgical & Orthopaedic Physiotherapy Screening Clinic and Multidisciplinary Service and returned to the specialist medical outpatient department (n = 3) or were urgently upgraded to a different category (n = 2). Five participants were discharged from the Screening Clinic and Multidisciplinary Service and removed from the waitlist in the telehealth group only.
		3. Safety.	3. No safety incidents reported.
		4. Costs.	4. Telehealth was more cost-effective (AUD$11 930) compared with fly-in, fly-out (AUD$13 699) with average cost per slot favouring telehealth (AUD$362), compared with fly-in, fly-out (AUD$415). Estimated 12-montly cost for telehealth = AUD$66 518 and fly-in, fly-out = AUD$76 384.
Cox, 2013 [47]; Cardio-respiratory	Feasibility and repeatability of conducting & supervising a submaximal exercise test (step test) via remote video-monitoring (telehealth) compared to a supervised clinical environment (F2F).	***Concurrent validity of:***	***Concurrent validity***
		Physiological responses to step test (SpO_2_, rating of perceived exertion, heart rate).	Physiological responses: No significant differences reported in SpO_2_, rating of perceived exertion or heart rate between the telehealth and F2F assessments. Oxygen desaturation <90% occurred in two participants during both assessments.
		***Feasibility:***	***Feasibility***
		Participant experience of and preference for telehealth vs F2F assessments.	Metronome acoustics were significantly poorer in the telehealth assessment (*P* = 0.006). No significant differences reported in participant ratings for interaction or comfort between the two assessments. System usability score mean = 85.6/100 (95% CI 79.8-91.5). Most participants did not have a preference in the supervision method (90%). 10% preferred in-person supervision.
Demmelmaier, 2010 [48]; Musculo-skeletal	Determine the risk factors screened by physiotherapists using telehealth in persons with prolonged back pain. To describe the nature, content & extent of the telehealth assessment.	***Utility of telehealth:***	***Utility of telehealth***
		1. Extent of risk factor screening by physiotherapists.	1. Five specific factors were screened during the consultations: spinal pathology/radiating pain, disability, sick leave, coping & negative beliefs. Three factors were not screened: pain intensity, expectations of duration of pain, depressive mood.
		2. Overall content of the consultations.	2. The mean length of telehealth consultations was 8.5mins. Most of this time was spent discussing; the pain problem, administrative issues, actions ie, booking an appointment time & physical activity with 10% of time spent on screening factors.
Eannucci, 2020 [49]; General	Understand the difference in satisfaction between telehealth and F2F physiotherapy consultations and understand the contributing factors to satisfaction based on age, gender or insurance.	***Utility of telehealth:***	***Utility of telehealth***
		1. Study population.	1. Study population: No statistically significant differences were found between the F2F and telehealth groups in the study population demographics.
		2. Satisfaction with service delivery.	2. No significant differences in satisfaction with service delivery were found between the groups overall. Participants rated the ability to schedule an appointment significantly higher in terms of satisfaction in the telehealth group compared to the F2F group (*P* < 0.001). Participants in the F2F group reported higher satisfaction in achieving their treatment goals compared with the telehealth group (*P* = 0.004). The relationship between insurance type and overall satisfaction was found to be significant (*P* = 0.001).
Exum, 2020 [50]; Cardio-respiratory	Explore telehealth in acute care settings during the COVID-19 pandemic and determine if telehealth technologies can help provide multidisciplinary team care to patients who have contracted COVID-19 in a hospital setting.	***Utility of telehealth:***	***Utility of telehealth***
		1. COVID team telehealth strategy.	1. Initial evaluations were completed via telehealth. Nurses provided in-room assistance while patients performed transfers, ambulation, and activities of daily living. Telehealth was ceased for complex cases and a request for direct contact made.
		2. Risk-benefit of isolation room care.	2. Communication with advanced practitioners helped determine the risks and benefits of direct contact with patients. Telehealth consults were performed where risks outweighed benefits of direct contact.
		3. Physiotherapy telehealth strategies including assessments monitoring heart rate, ratings of perceived exertion (Modified Borg Scale), biofeedback, team assessments to identify mobility and activity of daily living deficits.	3. Initial challenges of telehealth consultations included scheduling, care coordination, patient availability and development of a digital communication strategy. Barriers included nurse availability, patient availability & willingness and increased physical/environmental demand to complete telehealth assessments.
Funderskov, 2019 [51]; Palliative care	Determine feasibility of using telehealth in a specialised palliative care setting and determine health workers perspectives on barriers and facilitators to using these types of consultations.	***Utility of telehealth:***	***Utility of telehealth***
		Results of thematic analysis.	Four key themes emerged suggesting that video consultations mediate an active patient and relative involvement, mediates access to care, mediates room for co-operation between health care professionals and medicates technical device use. Physiotherapists felt that the use of video consultations made it easier to see physical changes in the person’s illness compared with a telephone consultation ie, noticing breathing difficulties and other changes.
Galiano-Castillo, 2014 [52]; Cancer care	Determine the level of agreement between telehealth assessments conducted by a caregiver and remote physiotherapist and traditional F2F assessments for patients with breast cancer at risk of lymphoedema.	***Concurrent validity and inter-rater reliability of:***	***Concurrent validity and inter-rater reliability***
			Overall e-CUIDATE telehealth assessment appears to be valid and reliable for measuring lymphoedema in breast cancer survivors.
		1. Arm circumference and volume measures.	1. Good to high levels of validity and inter-rater reliability for arm circumference and volume measures (MD = -0.11cm to 0.30cm, ICC = 0.81 to 0.98). Lowest reliability estimate was for distal circumferential measures on non-affected side.
		2. Total arm volume.	2. Good validity of total arm volume measures (overall MD = 44.20mL to 57.25mL, α reliability estimate 0.90 to 0.94). High levels of inter-rater reliability on the affected and unaffected sides (ICC/Rho = 0.81 - 0.89).
		3. Diagnosis of lymphoedema.	3. 16.7% of survivors were diagnosed with lymphoedema by the physiotherapist compared with 20% by e-CUIDATE with good levels of inter-rater reliability (Rho = 0.89).
Grona, 2017 [53]; Musculo-skeletal	Feasibility of delivering physiotherapy using telehealth via a remote present robot (RPR) and interprofessional team in a community with no local physiotherapist.	***Utility of telehealth:***	***Utility of telehealth***
		1.Assessment guided by physical therapist over telehealth and performed in-person by LNP included: Range of motion, neurological testing (light touch, reflexes), straight leg raise, strength.	1. Objective assessment completed; impression of diagnosis was mechanical lumbar dysfunction.
		2. Satisfaction survey.	2. Participant was ‘very satisfied’ and ‘somewhat confident’ with the method of assessment, stating it was ‘probably’ as good as F2F and would ‘most definitely’ recommend this method of assessment to a friend.
		3. Semi-structured Interview with LNP using thematic analysis.	3.Two themes emerged in the thematic analysis; the value of the interprofessional interaction (mentoring) and benefit of patient centred care (teaching capabilities and keeping the client in the local area limiting transportation and childcare).
Harland, 2017 [54]; Musculo-skeletal	Explore attitudes of primary clinical stakeholders; referring general practitioners and physiotherapists that work within the musculoskeletal focused PhysioDirect telephone-based triage service.	***Utility of telehealth:***	***Utility of telehealth***
		1. Responses of physiotherapists working in, and not working in, the telephone service.	1. Most physiotherapists working in and outside PhysioDirect thought patients would be better off speaking to a physiotherapist over the phone initially (73%-74%) rather than waiting for a F2F appointment, and that patients should be given a choice to use it (78%-86%). Most would recommend family/friends use PhysioDirect (52%-59%). Most disagreed that: using a phone service was a waste of time (66%); and those with a physiotherapy problem should speak with someone over the phone first (49%-55%).
		2. General practitioner responses to attitudinal question regarding PhysioDirect services.	2. Most general practitioners thought patients would be better off speaking to a physiotherapist over the phone initially for advice quickly rather than waiting up to 4 weeks (71%) and that the PhysioDirect service is a good idea (50%-76%). Many disagreed that lots of problems can be sorted out over the phone without ever having to see a physiotherapist F2F (44%). Most disagreed that it was a waste of time for patients to speak with physiotherapists over the phone (65%) and thought that everyone with a physiotherapy problem should speak to someone over the phone in the first instance (67%).
Hollinghurst, 2013 [55]; Musculo-skeletal	Economic evaluation (cost-consequence) of using PhysioDirect services for patients presenting with musculoskeletal related problems.	***Utility of telehealth:***	***Utility of telehealth***
			Little difference between the groups was observed.
		1. Resource use.	1. Many PhysioDirect participants had a F2F appointment (46%) and/or medical consult (35%). Mean consult time was 20mins longer for F2F group. Healthcare expenditure/ use was similar between groups, except for travel (F2F costs were higher).
		2. Costs and consequences – comparing costs from health care provider, patient, carer and productivity perspectives.	2. There was no significant difference in total mean costs per patient or primary clinical outcome between the two groups. The PhysioDirect group received their first assessment on average 27 d earlier but had lower satisfaction levels. QALY’s were higher in PhysioDirect group by 0.009 (95% CI -0.000 to 0.018).
		3. Cost utility analysis: comparing costs to the NHS using quality adjusted life-years.	3. There was a small extra cost when using PhysioDirect, offset by an extra gain in QALY. (0.88 probability that PD is cost-effective).
		4. Sensitivity analysis.	4. Sensitivity Analysis: When accounting for improved efficiencies and missing NHS cost and QALY, it appears the F2F is more expensive than PhysioDirect with a probability of cost-effectiveness for PhysioDirect = 0.72.
Hwang, 2016 [56]; Cardio-respiratory	Determine validity and reliability of performing video-based telehealth assessments including the 6-min walk test, timed up & go and grip strength compared with F2F assessments in participants with chronic heart failure.	***Concurrent validity and reliability of:***	***Concurrent validity and reliability***
			Overall, there was no significant difference found between telehealth and F2F assessments.
		1. Six-minute walk test and timed up & go test.	1. Good validity, inter-and intra-rater reliability for six-minute walk test (MD = -4.3m, 95% CI 0.74-0.96, ICC’s = 0.90-0.99) and timed up and go test (MD = 0.24s, 95% CI 0.64-0.94, ICC = 0.85-0.96).
		2. Grip strength test.	2. Good validity, inter-and intra-rater reliability for the grip strength test (MD = 0.16kg – 0.25kg, 95% CI 0.94-0.98, 0.94≤ICC>0.99).
		3. System usability scale.	3. Usability of the telehealth system was rated highly (mean score = 85/100).
		4. Technical issues encountered.	4. Technical issues encountered included internet dropouts (29%), auditory issues (12%) and visual clarity issues such as freezing and transmission delay (24%). 35% reported no technical issues.
Kinder, 2019 [57]; Women’s health	Describe the workflow of using an online platform for pelvic health physiotherapy and report participant perceptions of using telehealth.	***Utility of telehealth:***	***Utility of telehealth***
		Participant’s perceptions (14-questions survey including Net Promotor Score on general user experience regarding the software and satisfaction of the specific telehealth assessment).	Participant rated system as ‘very high quality’ and would recommend the system to others. The participant reported she would rather travel to see a physiotherapist for the initial visit rather than use telehealth. She felt ‘comfortable with the equipment used’ and was able to see and hear the clinician clearly. She reported her needs were met and received good care throughout the session.
Lade, 2012 [58]; Musculo-skeletal	Determine the validity, inter- and intra-rater reliability of performing a physical examination via telehealth compared with a F2F assessment for diagnosing elbow musculoskeletal disorders.	***Concurrent validity and reliability of:***	***Concurrent validity and reliability***
		1. Clinical diagnosis.	1. Exact agreement of pathoanatomical diagnosis was poor (validity 36%, inter-rater reliability 18%), but improved to moderate levels for similar agreement (73%). Good intra-rater reliability levels found for exact and similar agreement (73%-82%). Agreement levels for systems diagnosis were moderate to very good (validity, inter-rater and intra-rater reliability 64% - 90%).
		2. Assessments including: elbow range of motion, nerve tests, special orthopaedic tests, pain response, joint assessment and strength.	2. Validity was found to be good to very good for elbow range of motion, special orthopaedic tests, pain response and strength assessments (75%-90%). Decreased levels of validity were found for joint and nerve assessments (46%-47%). Good to very good levels of inter-rater reliability were found across all assessments (93%-98%) except for nerve assessments (68%). Intra-rater reliability across all assessment measures was found to be very good (81% - 98%).
		3. Limiting factor, severity scale and numerical analogue scale.	3. Validity, inter-rater and intra-rater reliability for limiting factor, severity scale and numerical analogue scale 68%-96%.
		***Satisfaction (VAS):***	***Satisfaction***
		Confidence in using telehealth, would they recommend to a friend, is it as good as in-person assessment, visual clarity, audio clarity and overall satisfaction measured.	Overall participants were satisfied with the telehealth assessment (mean >7/10 VAS) however the majority did not think the telehealth was as good as F2F (mean = 3.6 VAS).
Lovo, 2019 [59]; Musculo-skeletal	Examine the experiences of clients and staff operating in a team and technology model of care for people with chronic back disorders.	***Utility of telehealth:***	***Utility of telehealth***
		1. Satisfaction: Confidence in using telehealth, would they recommend to a friend, is it as good as in-person assessment, visual clarity, audio clarity, overall satisfaction measured and additional comments.	1. Most participants were satisfied with the telehealth assessment (68.4%), reported confidence with the video conference assessment (63.1%), would recommend it to a friend (78.9%) and found the visual and auditory clarity good (68.4%-78.9%). Only 42.1% reported the video conference assessment being as good as F2F.
		2. Thematic analysis of semi-structure interview with participants and health practitioners.	2. 4 key themes emerged in the interview:
			*Access to care for CBD:* Time saved with less driving and less time off work for travel, rural care provided with familiar practitioners, optimising existing health care spaces/buildings in rural communities and enhanced access to physiotherapy care.
			*Effective interprofessional practice*: Interprofessional communication and rapport, patient-centred care with two practitioners involved, effective teamwork, respectful interactions and capacity building.
			*Enhanced clinical care*: Holistic care with expanded service availability, expertise in CBD and access to previously unavailable experienced practitioner.
			*Technology*: Participants embraced the technology, camera was clear and easy to use but failed to function twice, an external speaker was required, those with hearing issues at times found it challenging. Confidence with technology was important and considerations should be made for older participants.
Mani, 2019 (First published July 2019) [60]; Musculo-skeletal	Concurrent validity, inter-rater and intra-rater reliability of performing a cervical spine physiotherapy assessment using telehealth compared with F2F for participants with non-specific neck pain.	***Concurrent validity and reliability of:***	***Concurrent validity and reliability***
		1. Pain intensity (VAS), deep neck flexor endurance and active range of motion: Flexion, extension, rotation, lateral flexion.	1. Overall good levels of validity and reliability reported for pain Intensity (MD = 0.90 VAS, ICC = 0.99), and deep neck flexor endurance (MD = -2.28s, ICC = 0.99). Good levels of validity and reliability reported for active range of motion in most directions (lateral flexion, rotation, extension) (MD = -1.0cm to -0.30cm, ICC’s 0.96-0.99) except for flexion (MD = 1.20cm, ICC 0.94-0.98).
		2. Disability measure with Northwick Park Neck Questionnaire (NPQ).	2. Very good validity, inter- and intra-rater reliability reported when administering the questionnaire via telehealth compared with F2F (MD = 0.11 NPQ score, ICC = 0.99-1.00).
		3. Sagittal neck posture (sagittal head tilt angle, cranio-cervical angle and shoulder angle) and combined neck movement.	3. All elements of the neck posture assessment showed fairly good levels of validity (MD = -0.32° to -0.96°) and very good levels of inter-rater and intra-rater reliability (ICC = 0.93-1.00). Moderate levels of agreement found for combined neck movements (inter-and intra-rater reliability 76.4%-78.5%, k = 0.53-0.57).
Mehta, 2020 [61]; Musculo-skeletal	Reliability of knee and wrist range of motion assessments conducted by an experienced clinician and a student clinician via telehealth compared with F2F.	***Reliability of:***	***Inter-rater and intra-rater reliability***
			Overall good reliability for all range of motion measures (experienced clinician rating higher than student).
		1. Joint range of motion of knee (flexion and extension) conducted by a student compared with experienced clinician.	1.Inter-rater reliability for telehealth knee flexion assessments was good (ICC = 0.94) with intra-rater reliability also good for the student and clinician (ICC = 0.95-0.98). Reliability levels for knee extension were less (ICC = 0.79-0.93).
		2. Joint range of motion of wrist (flexion and extension) conducted by a student compared with experienced clinician.	2. Good levels of inter-rater reliability were found for telehealth wrist flexion assessments (ICC = 0.90). Intra-rater reliability was higher for the clinician (ICC = 0.98) compared to the student (ICC = 0.82). Reliability levels for wrist extension were slightly less (ICC = 0.78-0.91) with the student recording a lower level of agreement.
Mukaino, 2020 [62]; Cardio-respiratory	Feasibility of using commercially available devices and applications for telehealth consultations.	***Utility of telehealth:***	***Utility of telehealth***
		1. Assessments completed via telehealth, time and use of onsite assistance.	1. Assessments included SpO_2_, vital signs and basic motor ability. On average a 3-min was required between commencement of the session and the first activity. 40% of participants did not have any experience operating the tablet, however only one participant required onsite assistance.
		2. Telehealth satisfaction.	2. Overall participants were satisfied with the quality of service provided (average = 4.4 ± 1.1, 5-point scale). They could hear clearly and easily talk to their health care provider and felt that it saves them time (average = 4.5-4.9, 5-point scale).
Nicola, 2018 [63]; Paediatrics	Investigate the feasibility and concurrent validity of performing a motor assessment on school-aged children using telehealth compared with F2F.	***Concurrent validity of:***	***Concurrent validity***
		1. The Movement Assessment Battery for Children - 2nd Edition (MABC-2) Components included: Manual dexterity, aiming & catching and balance.	1. The percentage of exact agreement was poor for manual dexterity, aiming and catching and balance assessment areas (15%-31.67%). High levels of agreement were found when these were calculated within 3 points (81.67%-90%). For the total test score, there was 31.67% exact agreement and 100% agreement within 3 points.
		2. Score categorization for the MABC-2.	2. Most children scored within the same category when comparing F2F and telehealth methods. Discrepancies between the methods were seen when determining categorization (3 children scoring 1-1.5SD below the mean on telehealth and 1SD above the mean F2F). Two children scored 1-1.5 SD below the mean in the F2F method compared with 1SD above during telehealth.
		***Satisfaction (4-point Likert scale):***	***Satisfaction***
		Participant satisfaction questionnaire.	Children generally liked playing games with the person on the iPad (Apple Inc, Cupertino, CA, USA) (mean = 3.12) and most thought the assessments took the same amount of time (64.7%). 39.2% wanted to do the next game with a person beside them and 15.7% wanted to use the iPad (Apple Inc, Cupertino, CA, USA). Children could hear (mean = 3.67) and see (mean = 3.88) the person on the iPad (Apple Inc, Cupertino, CA, USA).
O’Donovan, 2020 [64]; Musculo-skeletal	Discuss and evaluate the rapid implementation of a telehealth service model for clients with haemophilia during the COVID-19 pandemic.	***Utility of telehealth:***	***Utility of telehealth***
		1. Types of physiotherapy assessment completed via telehealth consultations during COVI-19.	1. The way physiotherapy consultations were performed changed due to COVID-19 (46% telephone, 27% video, 27% F2F). The types of assessment included gait assessment (thought adequate) and joint range of motion (in -person required for optimal evaluation).
		2. Participation rates during COVID-19, satisfaction and future telehealth engagement.	2. 45% of respondents participated in a telehealth consultation with most respondents very satisfied (53%) or satisfied (41%) with the telehealth consultation. 40% of respondents were interested in a remote annual physiotherapy assessment.
Palacin-Marin, 2013 [65]; Musculo-skeletal	Determine the level of agreement between telehealth and F2F assessments for participants with chronic low back pain.	***Concurrent validity and reliability of:***	***Concurrent validity, inter- and intra-rater reliability***
		1. Lumbar spine mobility: Lateral flexion, finger-to-floor distance.	1. Validity of lumbar spine mobility measures was good (Finger-to-floor MD = -0.5cm-0.3cm, lateral flexion MD = 0.73°, α = 0.75-0.99) as was the inter- and intra-rater reliability (ICC = 0.92-0.96, α = 0.93-0.96).
		2. Endurance (Sorensen test) and lumbar motor control (anterior straight leg raise measured with perceived rating on six-point scale).	2. Moderate levels of validity were found for the Sorensen test (MD = 9.22s, α = 0.80) with intra- and inter-rater reliability levels better (ICC = 0.92-0.94, α = 0.93-0.95). Good levels of validity and reliability were found for the active straight leg raise assessment (MD = -0.66 perceived effort rating, ICC = 0.93-0.95, α = 0.93-0.97).
		3. Disability (Oswestry Disability Index), pain (VAS), health-related quality of life and kinesiophobia (Tampa Scale of Kinesiophobia).	3. Disability, quality of life, pain and kinesiophobia measures all had good levels of validity (MD = -0.68-1.06 scale scores, α = 0.94-0.99).
Pearson, 2016 [66]; Musculo-skeletal	Describe variables determining participant acceptability of the PhysioDirect service and understand differences in participant experience between PhysioDirect and usual care groups.	***Utility of telehealth:***	***Utility of Telehealth***
		Thematic analysis of content including: expectations of the PhysioDirect service; PhysioDirect as an access point into physiotherapy; and acceptable and less acceptable features of the service.	Those with expectations of physiotherapy involving ‘hands-on’ treatment thought PhysioDirect could not meet their needs, however some changed their minds afterwards. Many participants thought of PhysioDirect as a first step to accessing treatment. Some felt PhysioDirect was not necessary, preventing access to ‘proper’ physiotherapy services. Reasons for not contacting the service included perceived cost of calls, opening hours, low expectations of physiotherapy, other priorities and seeking out private practice services. One patient did not attend the usual care physiotherapy appointment due to wait times. Acceptable features of the PhysioDirect Service included: it was quick, efficient, convenient, accessible, no travel or parking requirements, knowledge and self-management advice (allowing physiotherapy at home). Less acceptable features (not evident in the usual care group) included: ‘impersonal’ care, poor observation of non-verbal communication (increased difficulty explaining condition/pain), difficulty explaining movements, difficulty describing their problem in a way that could be correctly interpreted.
Peterson, 2018 [67]; Musculo-skeletal	To determine agreement in classification of participants with acute/subacute low back pain using a telehealth assessment compared with a F2F assessment and to determine participant satisfaction with the telehealth assessment.	***Concurrent validity of:***	***Concurrent validity***
		1. Treatment based classification system agreement of specific exercise, manipulation /mobilisation & stabilisation.	1. Overall agreement of treatment-based classification was moderate (68.1%) with inter-rater reliability also moderate (κ = 0.52, 95% CI 0.32-0.72). Intervention groups participants were classified into included: specific exercise (telehealth = 25.5%, F2F = 31.9%), manipulation/mobilisation (telehealth = 38.3%, F2F = 29.8%) and stabilisation (telehealth = 36.2%, F2F = 38.3%).
		2. Classification of centralization/peripheralization, aberrant movements, straight leg raise >91°, straight leg raise >10° asymmetry, straight leg raise large but <91° & active straight leg raise.	2. Agreement levels for classification (centralisation, peripheralisation, aberrant movements, straight leg raise) were moderate (48.9%-59.6%) except for straight leg raise >91° (35.1%).
		***Satisfaction (VAS)***	***Satisfaction***
		As good as F2F, would they recommend to a friend, connection with therapist, visual clarity and audio clarity measured.	Most participants agreed they would recommend the telehealth assessment to someone unable to travel (97%), the telehealth assessment was as good as a F2F assessment (56%), they were equally connected to the therapist for both sessions (66%) and they could always see and hear the therapist (83%-98%).
Prada, 2020 [68]; Neurological	Case report for patient with Charcot-Marie-Tooth post hand-surgery with suspected COVID-19 infection.	***Utility of telehealth:***	***Utility of telehealth***
		Assessments completed via telehealth.	Telehealth sessions commenced follow suspected diagnosis of COVID-19. Eight x 1-h sessions were conducted over a 4-week period. Assessment included strength and dexterity (thumb opposition test), timed functional tests ie, tying shoelaces in place for formal dexterity tests, and exercises that provided information on strength.
Richardson, 2017 [69]; Musculo-skeletal	Determine the validity, inter-rater and intra-rater reliability of performing a musculoskeletal assessment for participants with knee pain using telehealth compared with F2F.	***Concurrent validity and reliability of:***	***Concurrent validity, inter-rater and intra-rater reliability***
		1. Primary diagnosis agreement (structure involved) and systems diagnosis agreement.	1. Moderate levels of exact agreement for patho-anatomical diagnosis were seen (validity 67%, inter-rater and Intra-rater reliability 67%- 89%), which increased for similar agreement (validity 89%, inter-rater and intra-rater reliability 94%-100%). There were high levels of validity and inter-rater reliability for systems diagnosis agreement (94%) and moderate levels of inter-rater reliability (67%).
		2. Individual clinical findings (eg, pain, range of motion and medial collateral ligament testing).	2. Substantial levels of agreement were found for the individual clinical findings (%EA = 83%-98.8%) with inter-rater and intra-rater reliability almost perfect in some instances (94%-100% exact agreement).
		***Satisfaction (VAS):***	***Satisfaction***
		Confidence in telehealth assessment, would they recommend to a friend, is it as good as in-person assessment, visual clarity, audio clarity and overall satisfaction measured.	Participants were confident in the telehealth assessment method, could hear and see the therapists and would recommend it to a friend in most cases (60-80mm VAS), however did not feel it was as good as F2F (20-40mm VAS).
Russell (a), 2010 [4]; Musculo-skeletal	Validity, inter-rater and intra-rater reliability of performing a physiotherapy assessment using telehealth compared with F2F for participants with musculoskeletal non-articular conditions of the lower limb.	***Concurrent validity and reliability of:***	***Concurrent validity, inter-rater and intra-rater reliability***
		1. Primary diagnosis agreement (structure involved) and systems diagnosis agreement.	1. Moderate level of exact agreement (68%) and higher levels of similar agreement (79%) were found for pathoanatomical diagnosis. Reliability levels ranged from moderate to very high for exact and similar agreement (63%-100%). There was good agreement for systems diagnosis (79%) and very good inter-rater and intra-rater reliability (89%-100%).
		2. Individual clinical findings (ie, range of motion, swelling, gait, pain).	2. Good levels of agreement were found for the individual clinical findings (77.3%-82.9% exact agreement) with very good levels of inter-rater and intra-rater reliability (93%-97.4% exact agreement).
		***Satisfaction (VAS):***	***Satisfaction***
		Confidence in telehealth assessment, would they recommend to a friend, is it as good as in-person assessment, visual clarity, audio clarity and overall satisfaction measured.	Participants were confident in the telehealth assessment method, could hear and see the therapists and would recommend it to a friend in most cases (60-80mm VAS), however did not feel it was as good as F2F (20-30mm VAS).
Russell, 2013 [70]; Neurological	Determine the agreement, inter-rater and intra-rater reliability of conducting a physiotherapy assessment remotely using telehealth compared with a F2F assessment for participants with Parkinson Disease.	***Concurrent validity and reliability of:***	***Concurrent validity, inter-rater and intra-rater reliability***
		Timed up & go test, timed stance test, step test (right and left foot), steps in 360-degree turn (right and left), Berg Balance Scale.	The difference in scores for the timed up & go and timed stance test were MD = -0.01s (SD = 0.63), MAD = 0.47 and MD = 0.44s, MAD = 1.58 respectively. Limits of agreement for the timed up & go were -1.25 to 1.24 and timed stand test were -4.71-5.06. There were moderate levels of exact agreement for the step test (66.7%-75%), and steps in 360-degree turn (66.7%-75%) which improved when analysed as agreement withing one point (83.3%-100%, k = 0.95-0.98). There were poor levels of exact agreement for the Berg Balance Scale overall (16.7%, k = 0.94), which improved when analysed as agreement within one point (75%). Most individual elements of the Berg Balance Scale recorded high to very high levels of exact agreement or agreement within one point (75%-100%), many with perfect agreement except for standing on one leg (50%-83.3%). Intra-rater ICC’s for all assessment items were ≥0.96. Intra-rater ICC’s for telehealth assessments were ≥0.98.
Russell (b), 2010 [71]; Musculo-skeletal	Determine the criterion validity, inter-rater and intra-rater reliability of performing a remote musculoskeletal physiotherapy assessment of the ankle compared with a F2F assessment.	***Concurrent validity and reliability of:***	***Concurrent validity, inter-rater and intra-rater reliability***
		1. Primary diagnosis agreement (structure involved) and systems diagnosis agreement.	1. Moderate levels of exact agreement were found for validity and inter-rater reliability of primary diagnosis (46.7%-53.3% exact agreement) and high levels of exact agreement were found for intra-rater reliability (93.3%). Very high levels of similar agreement were found for validity and reliability (93.3%-100%) of primary diagnosis. High levels of validity, inter-and intra-rater reliability were found for agreement of systems diagnosis (80%-93.3%).
		2. Individual clinical findings (ie, range of motion, swelling, gait, pain).	2. Agreement for the individual clinical measures was consistently very high for reliability analysis (90.8%-99.9%) and more variable for validity analysis (76.4%-99.3%).
		***Satisfaction (VAS):***	***Satisfaction***
		Confidence in telehealth assessment, would they recommend to a friend, is it as good as in-person assessment, visual clarity, audio clarity and overall satisfaction measured.	Participants were confident in the telehealth assessment method, could hear and see the therapists and would recommend it to a friend in most cases (6-9cm VAS), however did not feel it was as good as F2F (>2cm VAS).
Salisbury, 2013 [72]; Musculo-skeletal	Determine the impacts of the PhysioDirect service on clinical effectiveness, acceptability, and access to care.	***Utility of telehealth:***	***Utility of telehealth:***
		1. Consultations (number, type and duration).	1. Most in the PhysioDirect group accessed the service at least once (85%) with 47% managed completely via PhysioDirect. The PhysioDirect group had fewer consultations (mean = 2.87) and shorter wait times (mean = 7 d) compared with usual care (mean = 3.25 and 34 d respectively).
		2. Clinical outcome at six months (36-Item short form survey, measure yourself medical outcomes profile, EQ-5D, global improvement score, pain, function and overall improvement).	2. There was no significant difference between groups in clinical outcome at the six-months mark.
		3. Quality of life, satisfaction and adverse events.	3. There was no difference in quality-of-life ratings between the two groups and no adverse events. Overall satisfaction was slightly less for the PhysioDirect group (mean = 75.9%) compared with usual care (mean = 79.7%). Both groups were equally satisfied with service access (69.1%-69.2%). 42%-51% of participants preferred usual care and 27%-40% preferred PhysioDirect.
Steele, 2012 [73]; Musculo-skeletal	Determine the validity, inter-rater and intra-rater reliability of using telehealth compared with F2F physiotherapy assessments to diagnose shoulder disorders and perform specific assessments and determine participant satisfaction of the telehealth assessment.	***Concurrent validity and reliability of:***	***Concurrent validity, inter-rater and intra-rater reliability***
		1. Primary clinical diagnosis agreement (structure involved) and systems diagnosis agreement.	1. Poor levels of validity and reliability were found for primary clinical diagnosis (18.52%-40.74% same agreement), improving for percent similar agreement (40.74-59.26). Good levels of validity (78.6%) and reliability were found for systems diagnosis (82.1%).
		2. Individual clinical findings (ie, range of motion, special orthopaedic tests, pain response to static muscle test, nerve range of motion, sensitisation, strength, joint assessment, pain, severity scale).	2. Good levels of validity were found for most assessments (75.9%-87.4%) except for nerve range of motion and sensitisation, joint assessment and limiting factor, where moderate agreement was found (56.1%-68.1%). Reliability was good for all assessment types (85.9%-98.3%) except nerve range of motion and sensitisation inter-rater reliability (66.9%). There was fair to substantial agreement for the validity of pain and severity (76.80%-96.00% exact and close agreement, k = 0.50-0.66) and almost perfect agreement for inter- rater and intra-rater reliability (97.20%-99.20% exact and close agreement, k = 0.83-0.95).
		***Satisfaction (VAS):***	***Satisfaction***
		How beneficial was the telehealth assessment, would they recommend to a friend, is it as good as in-person assessment, visual clarity, audio clarity and overall satisfaction measured.	Participants thought the telehealth assessment was beneficial, could hear and see the therapists and would recommend it to a friend in most cases (6-8cm VAS), however did not feel it was as good as F2F (3-4 VAS).
Truter, 2014 [74]; Musculo-skeletal	Determine the validity of performing a physiotherapy assessment of the lumbar spine via telehealth compared with a F2F assessment and determine participant satisfaction levels with telehealth.	***Concurrent validity of:***	***Concurrent validity***
		Assessment of posture in coronal/sagittal planes (symmetry, scoliosis, pelvic tilt, spinal asymmetry, pelvic position, lumbar lordosis, thoracic kyphosis & thoracic position), straight leg raise, active movement (flexion, extension, lateral flexion, rotation) and agreement direction was painful.	Poor to moderate levels of exact agreement found for coronal assessment of symmetry, pelvic tilt, presence of scoliosis and spinal asymmetry classification (35%-72%) and sagittal assessment of lumbar lordosis, thoracic kyphosis and pelvic position (25%-50%). Improved levels of exact agreement found for pelvic tilt, pelvic position and thoracic position (67%-75%). The correlation coefficient for active range of motion assessments were worse for lateral flexion (*r* = 0.67-0.69) and better for flexion and extension (*r* = 0.83-0.89). There was moderate correlation for straight leg raise measures between the two assessments (*r* = 0.64) and a high level of agreement that a direction was painful (81%-96% exact agreement). High levels of agreement found for symptoms with straight leg raise such as pain and sensitivity (82%-90% exact agreement), moderate levels of agreement for the reason for limits in movement (55% exact agreement, κ = 0.37) and worse movement direction (65% exact agreement, κ = 0.56).
		***Satisfaction (VAS):***	***Satisfaction***
		Confidence in telehealth assessment, would they recommend to a friend, is it as good as in-person assessment, visual clarity, audio clarity and overall satisfaction measured.	Participants were confident in the telehealth assessment method, could hear and see the therapists and would recommend it to a friend in most cases (60-90/100mm VAS), however did not feel it was as good as F2F (20-40mm/100 VAS).
Turner, 2018 [75]; Musculo-skeletal	To describe three case studies which conducted initial physiotherapy assessments via synchronous telehealth using the McKenzie Method of Mechanical Diagnosis.	***Utility of telehealth:***	**Utility of telehealth**
		1. Patient: results of mechanical assessment and classification (active range of motion, repeated movements, and/or sustained postures) and key telehealth assessment details.	1.The physiotherapist observed changes in posture, active range of motion, strength and pain across the case studies. All participants required some consultation time to alter the viewing position of their device or set up their device. They had ironed out any issues by the second telehealth consultation (participant two purchased a phone holder to improve viewing).
		2. Satisfaction rating.	2. Patients responded to emails at either one/three months post consultation and were ‘pleased’, or very satisfied with the virtual assessment. It was convenient, prevented travel and they liked the ease of use, clear explanations and knowledge of the clinician.
Wood, 2017 [76]; Cardio-respiratory	Evaluate health outcomes of adults with Cystic Fibrosis participating in a telehealth clinic and report participant uptake and satisfaction levels.	***Utility of telehealth:***	***Utility of telehealth***
		1. Uptake of telehealth.	1. 100 appointments attended by 21 participants. 66% via telehealth (19% completed all appointments via telehealth). Attendance increased to 90% compared with 19% the previous year. Mean attendance increased significantly from 2 to 5 (*P* < 0.001).
		2. Participant satisfaction: Telehealth Satisfaction Scale and a purpose-built survey (5-point Likert scale).	2. Respondents rated most items as ‘good’ or ‘excellent’ on the satisfaction survey. One participant rated voice quality of equipment as poor. 94% of participants felt telehealth clinics were a good way to manage their care, felt it met their expectations and felt they could communicate effectively using the technology. 41% were unsure or did not prefer telehealth clinics over F2F.
		3. Healthcare utilisation.	3. 80% of exacerbations detected were identified over telehealth. There was a significant increase in intravenous antibiotic days, hospital admissions and admission days per participant (*P* < 0.05).
		4. Spirometry, body mass index and Health-related quality of life (Cystic Fibrosis Questionnaire) and changes throughout use of the telehealth clinic.	4. No significant changes were seen in spirometry or body mass index. Improvement in the vitality domain of the Cystic Fibrosis Questionnaire (*P* = 0.04) was observed.
Wootton, 2020 [77]; Cardio-respiratory	Discuss the rehabilitation model developed for three patients with COVID-19.	***Utility of telehealth:***	***Utility of telehealth***
		Key elements of the telehealth service including 5-repetition and 1-min sit to stand tests and overall experience survey.	Each participant underwent an initial assessment with oxygen saturation levels and heart rate monitoring. Oxygen desaturation ≥3% triggered a medical review and cessation of activity. 5- repetition sit-to-stand and 1-min sit-to-stand were conducted via video conference (full view of participant) with a chair positioned against a wall. Dyspnoea, fatigue and other symptoms were monitored. Persistent dyspnoea for one participant triggered a medical review and diagnosis of a pulmonary embolism. Participants reported; improved confidence in knowing what to do during their recovery, benefits of support as they transitioned out of isolation, access to MDT education and symptom management.

F2F – face-to-face, LNP – licensed nurse practitioner, VAS – visual analogue scale.

### Areas of practice

**Figure 2:**
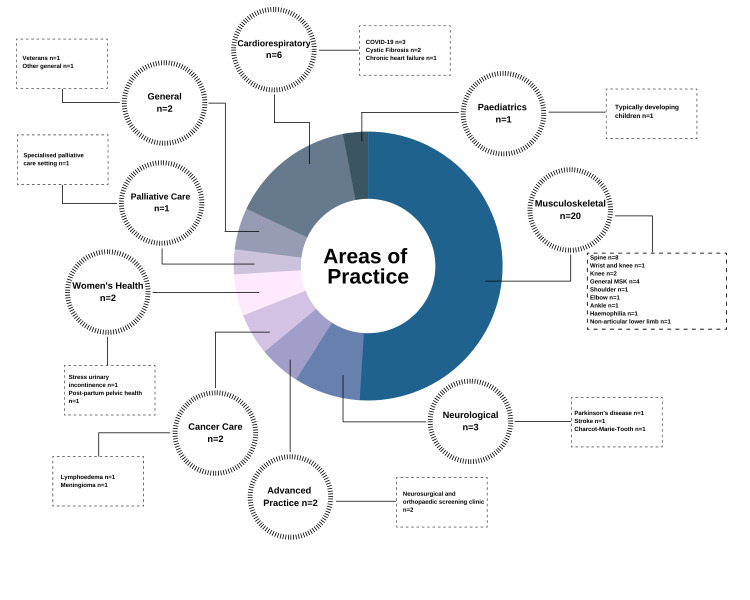
Physiotherapy practice areas of included studies and breakdown of population types for those studies with the largest representation.

The validity, reliability and utility studies included in this review were from a variety of physiotherapy practice areas (**Figure 2**). Four of these studies reported on the same ‘PhysioDirect’ (PD) telephone screening service and included participants presenting with a range of musculoskeletal conditions [54,55,66,72].

### Telehealth assessment environment

**Figure 3:**
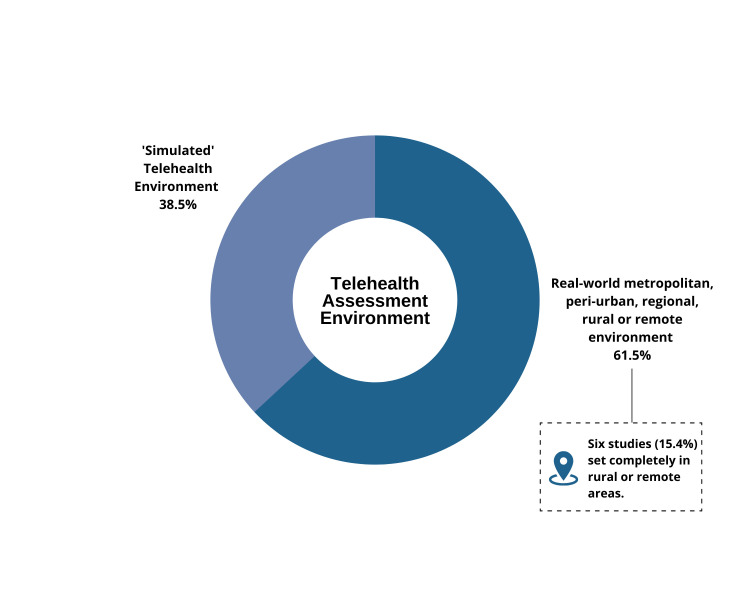
Environments where telehealth assessments were conducted.

The type of environment in which the telehealth assessment occurred was either ‘simulated’ (n = 15), which consisted of a university or clinic room set up for telehealth with the physiotherapist located in another room of the same building as the client, or ‘real-world’ (n = 24), where the client was located in a setting such as their home, school or second clinical environment and the physiotherapist was located elsewhere (**Figure 3**).

### Utility of telehealth assessments

#### Overall utility

Aspects of telehealth utility explored across the studies in this review (**Table 5**) included telehealth assessments in rural and remote environments, satisfaction and attitudes of clients and health care professionals towards telehealth, clinical outcomes, consultation and assessment types completed, telehealth strategies employed, service access, and technology and economic evaluation. The studies which considered aspects of telehealth utility were set across a broad range of physiotherapy settings, including home, community, and hospital settings.

Nine of the utility studies were case studies or descriptive papers, with four of these reporting on telehealth use during the COVID-19 pandemic [50,64,68,77]. Despite some initial challenges with scheduling and rapid adoption of a new method of service provision, telehealth physiotherapy services for participants diagnosed with COVID-19 facilitated and maintained service access at times when isolation was required. One study reported 73% of physiotherapy consultations moved to telehealth (46% telephone, 27% video conference) during the COVID-19 pandemic [64]. Strategies used to provide the telehealth assessments to patients with COVID-19 included delivery of pulse oximeters to the patient’s home [77], development of a triage system, utilisation of nursing staff already in contact with the patient in isolation to assist with telehealth assessments [50], and use of functional tasks in lieu of formal assessment items to infer hand strength and dexterity following hand surgery [68].

One observational study [48] and the remaining case studies/series included in this review, investigated the use of telehealth assessments for participants with either low back pain [48,53,75], gender health concerns (pelvic dysfunction or post-partum support) [44,57] or cancer care [42]. Of these studies, one study reported telehealth consultation lengths ranging from 3-21 minutes (mean 8.5 minutes) with consultation time devoted to pain, administrative issues, and actions, ie, bookings and advice on physical activity [48]. The value of interprofessional practice, including ability to mentor, train and provide patient-centred care was recognised, along with the benefits of decreased travel time and childcare requirements [53].

Taken together, these studies indicate some feasibility of using synchronous physiotherapy telehealth assessments in a range of areas of physiotherapy, suggesting it can provide benefits to the community and help to maintain or establish service provision when physiotherapy presence or expertise is lacking or when disease transmission is of concern.

#### Utility of telehealth assessments in rural and remote environments

Telehealth assessments occurring in rural and remote areas (**Figure 3**) were offered because physiotherapy services in the region were lacking or participants required consultation with an experienced physiotherapist in a specialised area of practice. These studies were conducted in various countries throughout the world with participants including those with cystic fibrosis (CF) [76], neurosurgical and/or orthopaedic concerns [46], chronic back disorders [53,59], stress urinary incontinence [44] or veterans with varying concerns [10]. Comparisons between synchronous telehealth assessments and face-to-face physiotherapy assessments for fly-in, fly-out services [46] or specialist centre appointments [76] were investigated. Two studies used nurse practitioners as assistants [53,59] with one of these also using a remote presence robot (RPR) (51) to perform the remote physiotherapy assessment. These studies reported a variety of findings which included: offering a telehealth clinic increased attendance rates [76], participants felt satisfied [44,53,59,76] and confident [59] with the telehealth assessment most of the time, expectations of the assessment were met via telehealth, and that it appears to be cost-saving when compared with a fly-in, fly-out service [46].

Key themes from patients and health care providers using telehealth consultations across rural areas included: that telehealth enhanced access to care (particularly for those in underserved areas), was more convenient, promoted interprofessional communication and team functioning [10,59] and appeared safe with no reports of safety incidents [46]. Having a licensed nurse practitioner on site during the telehealth physiotherapy assessment allowed for identification of unexpected issues, was capacity building and helped improve holistic care of individuals [10,59]. Challenges identified by those participating in telehealth consultations in rural areas included: the consent procedures look longer; a lack of access to equipment/specialised equipment in the home setting; using unfamiliar technology; viewing and positioning of the camera; poor quality video; and problems with data file size [10].

#### Satisfaction and acceptance

Client and health care practitioner perceptions regarding acceptability and/or satisfaction with physiotherapy telehealth assessments were reported in 19 of the included studies, with most stating they were satisfied with the telehealth assessment and consultation process. Nine studies conducted similar satisfaction surveys [4,45,58,59,67,69,71,73,74], with questions asked rated on a visual analogue scale (VAS). The questions included: 1) level of confidence in, or how beneficial they rated the online assessment, 2) would they recommend a telehealth assessment to a friend who is unable to travel, 3) is telehealth as good as face-to-face appointments, 4) visual clarity, 5) audio clarity, and 6) overall satisfaction [4,45,58,67,69,71,73,74]. Most questions asked rated highly in each of these nine studies (>6/10 VAS). If given the choice, many preferred the face-to-face method of assessment. This trend was also seen in another study [76], indicating high levels of agreement that the timing, organisation, communication, and overall expectations were met using this service delivery method, but most preferred face-to-face appointments if available.

A participant satisfaction questionnaire specifically developed using age-appropriate language was completed with children who participated in the feasibility and validity study of telehealth physiotherapy services conducted by Nicola and colleagues [63]. Participants appeared to like using the iPad (Apple Inc, Cupertino, CA, USA) (mean >3), however, only 15.7% of children would pick ‘playing the next game’ (assessment) on the iPad (Apple Inc, Cupertino, CA, USA), with 39.2% wanting to play with a person beside them and 45.1% happy to do either.

Ratings of participant and/or clinician experience and satisfaction with telehealth physiotherapy assessments during the COVID-19 pandemic were reported in three of the included utility studies in this review [49,62,64]. These papers reported overall good levels of satisfaction with telehealth assessments, one reporting no statistically significant difference in satisfaction levels between face-to-face, telehealth or combined types of services and a higher satisfaction level for participants in the telehealth group for appointment scheduling (*P* < 0.001) [49]. One study reported mean overall satisfaction levels of 4.4 ± 1.1 on a 5-point Likert scale, reported participant perceptions of better access to health care services using telehealth (mean = 4.9 ± 0.3), and found participants believed telehealth saved them travel time (mean = 4.9 ± 0.3) and allowed them to easily talk to their health care provider (mean = 5) [62]. The third study reported that participants with haemophillia were ‘interested’ or ‘very interested’ in participating in a remote annual physiotherapy assessment, with 41% of participants satisfied and 53% very satisfied with their telehealth consultation [64].

Healthcare provider and participant acceptance was reported to varying degrees across three of the studies investigating the PhysioDirect (PD) telehealth service compared with usual care (UC) in the United Kingdom (UK) [54,66,72]. 44% of physiotherapists working in the PD telehealth service and 50% of physiotherapists working outside of the PD telehealth service disagreed/strongly disagreed that many physiotherapy problems can be managed without ever having to see a physiotherapist face-to-face. However, over 73% of physiotherapists agreed that clients would be better off speaking with a physiotherapist over the phone rather than waiting for a face-to-face appointment, with many reporting they would encourage a family/friend with a physiotherapy related issue to contact PD services in the first instance [54]. UC physiotherapy consultations rated significantly higher in terms of satisfaction when compared to telehealth [72] and the acceptance of a telephone physiotherapy consultation service was variable, with some participants commenting on the ‘hands on’ nature of physiotherapy not able to be accomplished over the phone and that the PD service impaired access to ‘proper’ physiotherapy services [66]. The features of the PD service that appeared to be acceptable to participants included convenience, access to a helpful and knowledgeable physiotherapist and that it fostered delivery of self-management advice. The PD service was reported by some as ‘impersonal’, with communication difficulties impacting service quality [66].

#### Technology and usability

The video conferencing and telephone-based technologies used to perform telehealth assessments are listed in **Table 4**. The most common were telephone telehealth systems (used in ten studies) and the ‘eHAB telerehabilitation system’ (NeoRehab, Brisbane, Australia) which was used in nine. A variety of laptop and desktop computer systems was used, along with wall-mounted, external or embedded cameras. Some papers required participants to use their own smart device (ie, iPad, tablet, phone) during the telehealth consultations, whilst others had telehealth assessment equipment set up in a room ready for the consultation. Additional peripherals such as pulse oximeters, LCD screens, tripods, portable speakers and microphones were used in various studies. Several studies reported inconsistencies in technology as limitations, which included: low/fading audio and sound quality [47,56,58,70]; voice disconnection [60]; problems with video quality [56,58]; communication failures [69]; image resolution issues [74]; freezing of live images [60,71] and other technical problems not specified [52]. The usability of telehealth to complete physiotherapy assessments was reported in two studies [47,56] which used the System Usability Scale [78] as an outcome measure. Both studies reported positive responses (scores ≥85 out of a possible 100) and patients seemingly willing to adopt the telehealth technology.

#### Challenges encountered during telehealth assessments

Although there were reports that participants ‘embraced’ the technology used in the telehealth assessments [59], several challenges impacted the effectiveness of telehealth assessments in the included studies. Teaching participants to accurately perform and report some assessment items, for example orthopaedic tests, was challenging [71]. Communication was also challenging, as those participants less experienced in video conferencing etiquette appeared more reserved in their conversation as they attempted to take in information. Some participants had more difficulty explaining and comprehending information [71] or did not stay in the range of the microphone. Others did not wish to de-robe during the telehealth consultation for a variety of reasons, including cultural reasons and this may have impacted upon the visibility of posture and results of range of movement assessments [74].

#### Economic impact

The economic impact of utilising telehealth assessments in place of face-to-face assessments was investigated in two utility studies included in this review. One study involved a cost audit comparing a video conference telehealth physiotherapy service with a fly-in, fly-out physiotherapy service [46]. Telehealth appeared to be a cost-effective option for rural services, with an estimated 12-month cost of AUD$66 518.00 compared to AUD$76 384.00 for a fly-in, fly-out service [46]. The other study compared cost-effectiveness of a telephone-based telehealth physiotherapy service (PD) with usual face-to-face care (UC) [55]. The authors found the difference between the PD and UC groups was minimal in terms of cost, finding PD is probably more cost-effective if the physiotherapist’s time is productive. However, underutilisation of the service increased costs of the PD service slightly compared with UC, which were then offset by improvements identified through health-related quality of life questionnaires (quality adjusted life years).

### Validity and reliability of telehealth assessments

The assessment tools investigated across the validity and reliability studies included in this review and the results of those studies are summarised below, with details provided in **Table 5**. The types of assessments completed by physiotherapists via telehealth were either pre-determined or decided upon by the assessing clinician at the time of the assessment. Such discretion was apparent in six of the validity and/or reliability studies [4,45,58,69,71,73], where telehealth clinicians could decide which assessment measures to use. These studies all reported on the validity and reliability of clinical diagnosis (primary or pathoanatomical, and systems diagnosis) and management decisions rather than specific assessment tools, except for one study [58] which reported on both.

#### Validity of telehealth assessment

Assessments of the upper limb performed using synchronous forms of telehealth that were associated with good or very good levels of concurrent validity when compared to a face-to-face assessment were (**Table 5**): assessments for participants with elbow [58] or shoulder [73] pathology inclusive of range of motion (ROM), special orthopaedic tests, pain response and strength (75%-90% agreement); diagnosis and management decisions of shoulder pathology (83.3% exact agreement) [45]; pathoanatomical diagnosis of the elbow (73% similar agreement) [58]; systems diagnosis of shoulder and elbow pathology (73%-78.6% agreement) [58,73]; and circumferential measures taken by a caregiver via telehealth under the direction of a physiotherapist to determine total arm volume for patients following breast surgery (largest mean difference occurring in circumferential measures taken 10cm below the elbow, MD = 0.28cm-0.30cm) [52].

Synchronous telehealth physiotherapy assessments of the lower limb associated with good or very good levels of concurrent validity when compared to a face-to-face assessment included (**Table 5**): clinical diagnosis of knee and ankle pathologies (exact or similar agreement in 83.3% - 93% of cases) [45,69,71]; systems diagnosis for knee or ankle pathologies (≥80% observed agreement) [69,71]; and primary clinical diagnosis (79% similar agreement) and systems diagnosis (79% observed agreement) of non-articular lower limb injuries [4]. Good levels of concurrent validity were also found for assessments of the cervical and lumbar spine, including (**Table 5**): ROM [60,65,74]; active straight leg raise [65]; lumbar spine assessment of painful direction of movement [65]; neck pain intensity [60]; deep neck flexor endurance [60]; cervical spine posture [60]; questionnaires to determine levels of disability, quality of life and pain [65]; and diagnosis and management decisions (including decisions about necessary investigations) for pathologies resulting in lumbar spine symptoms (83.3% exact agreement) [45].

Assessments that required observation and/or timing, such as the timed-up-and-go test (TUG), timed stance test, step test and steps in 360-degree turn, also showed good levels of concurrent validity when compared with face-to-face assessments [47,56,70], as did the six-minute walk test (6MWT) and grip strength test, which were both reported as possessing concurrent validity when performed via telehealth in participants with chronic heart failure [56] (**Table 5**).

Some assessments conducted using synchronous telehealth methods were standardised physiotherapy assessments such as The Movement Assessment Battery for Children (2^nd^ edition) (MABC-2) assessment conducted in typically developing children [63], the ABILHAND assessment conducted in participants following stroke [41] and the Berg Balance Scale for participants with Parkinson disease [70]. These assessments were found to have higher levels of concurrent validity when compared to face-to-face assessment if scores were either analysed within a certain number of points, or totaled. The level of validity decreased when looking at exact agreement or individual items within each assessment tool, such as standing on one leg (50% exact agreement) or threading a needle and fastening a snap (k coefficients 0.15 and 0.20 respectively).

Moderate to good levels of exact agreement were found for primary clinical diagnosis and systems diagnosis of non-articular lower limb injuries [4]. Moderate levels of concurrent validity were also found for cervical flexion and combined movements of the cervical spine [60]; and overall pathology classification decisions made by assessors (68.1%) for participants with low back pain [67], with a significant difference found between the telehealth and face-to-face assessments for straight leg raise classification greater than 91° [67].

Poor to moderate levels of concurrent validity were found for upper limb nerve testing and sensitisation assessment [58,73], joint assessment of the elbow and shoulder (46%-64.4% agreement) [58,73], pathoanatomical diagnosis of the shoulder (59.72%) [73], the Sorenson test [65], and postural assessments investigating symmetry, pelvic tilt and spinal asymmetry in the coronal plane [74]. Additional details are provided in **Table 5**.

#### Inter-rater reliability in telehealth assessment

Good to very good interrater reliability was reported for synchronous telehealth assessments involving the upper limb, including (**Table 5**): elbow and shoulder ROM; upper limb special orthopaedic tests; strength assessments; elbow and shoulder joint assessments; pain; and pain response to static muscle tests (87%-98.3% agreement) [58,73]. Lymphoedema assessment measures of the upper limb such as arm circumferential measures and calculations of total volume were reported to have high levels of inter-rater reliability (ICC 0.81-0.98) [52]. Good to very good levels of inter-rater reliability were found for lower limb assessments, including: knee ROM [61]; knee swelling [43]; and diagnosis of knee pathology [69], non-articular lower limb injuries [4] and ankle disorders [74]. Assessments of the cervical and lumbar spine [60,65], including ROM, lumbar spine mobility, lumbar spine motor control, posture, and muscular endurance, also showed good to very good inter-rater reliability (ICC 0.93 – 0.99). Assessments such as functional strength testing (30-second chair-to-stand test), TUG, and gait assessment (Tinetti test) displayed good inter-rater reliability (0.79≤α≥0.87) [43], as did the 6MWT, timed stance test, step test, steps in 360-degrees turn, grip strength test, and Berg Balance Scale (all ICC>0.96, α = 0.76 for Berg Balance Scale) [43,56,70].

Moderate to good levels of inter-rater reliability were reported for primary diagnosis of upper limb disorders (73% agreement elbow and shoulder) [58,73] and assessing the cervical spine using combined neck movements (78.5% agreement) [60].

Poor to moderate levels of interrater reliability were reported for upper limb nerve tests involving nerve ROM or assessment of neural sensitisation (66.9%-68% agreement), and systems diagnosis of elbow disorders (64% agreement, *P* = 0.11) [58,73]. Inter-rater reliability for the assessment of scars was poor (α = 0.34) with authors suggesting that still photographs may be better than synchronous telehealth via video conference [43].

#### Intra-rater reliability in telehealth assessment

Good to very good rates of intra-rater reliability were reported for assessment of the elbow (81%-98% agreement) [58], shoulder (% agreement >85%) [73], cervical spine (ICC>0.93) [60], and lumbar spine using ROM, endurance and motor control assessments (ICC>0.94) [65]. Very good levels of intra-rater reliability were found for the diagnosis of knee pathology [69], non-articular lower limb injuries [4], ankle disorders [71], elbow [58] and shoulder disorders [73]. Very good levels of intra-rater reliability were reported for knee and wrist flexion/extension ROM assessments conducted by experienced clinicians with healthy participants, however levels decreased when student physiotherapists performed the same measures [56,61]. Intra-rater agreement was high for the TUG, 6MWT, timed stance test, step test, steps in 360-degrees turn, grip strength test and Berg Balance Scale (ICC≥0.96) [70].

## DISCUSSION

The review identified a total of 39 studies published over the past decade which have investigated the validity, reliability or broader utility of using synchronous telehealth physiotherapy assessment methods across MSK, neurological, cardiorespiratory, paediatric, women’s health, cancer care, palliative care, advanced practice and general fields of physiotherapy practice. Just over 60% of studies published were in a real-life community, hospital or home setting, with six of these papers set in a rural or remote area that faced limitations in access to physiotherapy services. The major findings of this review demonstrate that performing many types of physiotherapy assessments via synchronous forms of telehealth appears to be valid and reliable, with those more observational in nature more easily performed and having higher levels of validity and reliability. Participant satisfaction levels with telehealth assessments were generally high, although when asking participants if they would favour or select a telehealth assessment compared with a face-to-face assessment, most would prefer the latter. The utility of telehealth amongst physiotherapy areas of practice is expanding, with increased perceptions of its acceptability among health care professionals and recipients of care when service access is a problem. Research within this area is also growing, with approximately two thirds of included studies published in the last 5 years, and later studies adapting their telehealth assessment techniques based on the findings and issues reported by earlier researchers.

### Areas of practice

To our knowledge, this is the first systematic review that has investigated the validity, reliability and broader utility of performing physiotherapy assessments using synchronous forms of telehealth, across all areas of physiotherapy practice. The current review considered a broader range of physiotherapy assessment tools than have been previously considered. For example, a systematic review investigating the validity and intra-and inter-rater reliability of telerehabilitation (TR) physiotherapy assessments for patients with specific musculoskeletal disorders was reported in 2016. That review, conducted by Mani and colleagues [19] identified eleven studies that met eligibility criteria and included both synchronous and asynchronous forms of assessment. A more recent review by Grona and colleagues [25], also in the field of musculoskeletal physiotherapy, investigated video conferencing consultations, including both assessment and intervention aspects of the telehealth service. Seven of the studies that were included in both of these previous systematic reviews also met the eligibility criteria of our systematic review. Neither of these previous systematic reviews investigated all synchronous forms of physiotherapy assessments used across all areas of physiotherapy practice.

Many areas of physiotherapy clinical practice do not have enough presence in the literature to enable accurate determination of the validity and reliability of relevant synchronous telehealth assessments. More than half of the papers in this review focused on assessments used in the field of musculoskeletal physiotherapy. To address issues of access through approaches that include telehealth assessment, it is important to investigate the clinometric properties of assessment tools used commonly in all areas of physiotherapy practice. The results of our review show that several areas of physiotherapy practice are completely lacking in research regarding the validity, reliability and utility of telehealth assessments. There were no studies meeting our inclusion criteria that investigated physiotherapy assessments with infants or with children of any age with existing difficulties in gross motor function. This population arguably would benefit greatly from telehealth assessments performed within the home setting, not only to ease the travel burden for families and improve access, but also to gain a better insight into the child’s function in the home environment. Children feel more comfortable and display a greater repertoire of skills when in their home environment, as compared to that of a physiotherapy clinic [79]. There were also no studies that examined the validity and reliability of synchronous telehealth assessments in the clinical areas of elite sport, gerontology, occupational health, men’s health or other specialty areas of physiotherapy practice, including hand and orofacial physiotherapy. Only a very small number of validity and reliability studies, investigating assessments relevant to limited types of health conditions, were identified in cardiorespiratory, paediatrics, chronic pain, neurological and cancer care (particularly lymphoedema) physiotherapy. It could be argued that individuals requiring physiotherapy consultation in these less represented fields of physiotherapy may benefit the most from having valid and reliable telehealth assessments, given the need for specialised services (which may not be present in regional, rural or remote areas) or limited ability to travel due to illness. Individuals requiring a physiotherapy assessment for conditions that fit within these areas of practice (for example, those that have existing cardiorespiratory conditions, are immunocompromised, or older adults), are also those classified in the ‘highest risk’ groups for COVID-19 and would at times require telehealth rather than face-to-face consultations, particularly when disease transmission is of concern [80]. More comprehensive research in these areas of physiotherapy practice is required.

### Telehealth assessment environments

Many validity and reliability studies included in this review were set in a ‘simulated’ telehealth environment and therefore aspects of their findings require cautious interpretation. The environment in which telehealth assessments are performed can impact the assessment quality, with aspects such as lighting, image resolution and internet speed important for visualisation using synchronous video-based telehealth assessment. It may also influence participants’ ratings of satisfaction and acceptance of the method of assessment, as access to technologies and knowledge that a clinician is close-by in a simulated environment could be reassuring.

Some of the case studies completed in hospital environments that described changes in practices for consultations during COVID-19 did, however, require a telehealth assessment to occur despite the clinician and participant being collocated in the same building. Due to the infectious nature of the disease, it was important to keep contact between physiotherapist and participant limited to decrease potential virus transmission. In this circumstance, the results of previous studies conducted in ‘simulated’ environments is very helpful in guiding the set up and execution of these types of telehealth services.

Nevertheless, many of the utility studies included in this review were conducted in ‘real-life’ environments where the participant was located in an environment representative of what would occur outside research.

### Utility of telehealth assessments

The use of telehealth in the clinical setting has increased dramatically over the past 12 months, with many clinicians compelled to use telehealth in one form or another during the COVID-19 pandemic. Four of the studies included in this review evaluated or described how clinicians had transitioned to telehealth for maintaining physiotherapy services for their patients [50,64,68,77]. Prior to COVID -19, the use of telehealth was seen as a way to reduce barriers to accessing services, such as distance, inability to travel, or unavailability of a physiotherapist in the local area [7]. Throughout the COVID-19 pandemic, we have seen the value of telehealth change, with physiotherapists completing synchronous physiotherapy assessments while located within the same hospital as the patient, and at times, just outside a patient’s room [50]. This change in the way we utilise telehealth services in the ‘real-world’ encourages us to consider more closely and perhaps increasingly value findings of studies that have been conducted in ‘simulated’ environments where the physiotherapist and participant are collocated in the same building. The way in which specific assessments were performed via telehealth also needs to be considered when interpreting the results. Some studies did not include a detailed description of how the assessments were performed or adapted to meet telehealth requirements, and others used non-standardised, functional activities to assess underlying impairments.

#### Utility of telehealth assessments in rural and remote environments

Six utility studies were conducted with participants located in rural and remote geographical areas, providing insight to the challenges and benefits of telehealth assessment. Due to the relatively small number of studies set in these geographical areas, it is important that we see further research investigating synchronous telehealth physiotherapy assessments set in ‘real-life’ rural and remote settings. Many other studies were completed in ‘real-life’ environments but located in more urban and metropolitan areas.

#### Satisfaction and acceptance

The level of satisfaction and acceptance of physiotherapy telehealth assessments amongst telehealth users was generally high, with satisfaction measured in several studies, across a range of environments. Most participants thought telehealth would be useful for those unable to travel to or access a suitable physiotherapist in their local area but would not prefer it over a face-to-face appointment. This finding is consistent with previous research investigating patient satisfaction using synchronous and asynchronous forms of telehealth in medicine, which reported that telehealth can provide a high-quality service, increase access to care, decrease travel time and empower patients to manage their chronic conditions, but can be met with resistance from those who have difficulty embracing change [81].

#### Technology and telehealth usability

There were several studies in this current review that identified inconsistencies in technology as limitations in physiotherapy telehealth assessment [47,52,56,58,60,69-71]. Some users were able to easily troubleshoot these issues or bypassed the synchronous telehealth system, for example by positioning a metronome with the participant rather than the remote physiotherapists when conducting a step test [47] and some provided recommendations for future studies, such as using still images rather than a live video feed to assess scars [43]. As technology advances and becomes more readily available within the community, there appear to be improvements in levels of digital literacy with an increased proportion of the population classified as ‘digitally native’, having grown up with access to technologies and having subsequent competence in navigating those that are new [82]. This may change the levels of acceptance and usability of technologies such as telehealth in the future and further limit inconsistencies identified in this systematic review. Consequently, it would be advantageous to consider developing further research studies within this topic area with the latest technology, for example 4K HD Cameras, inclusive of larger sample sizes.

#### Challenges encountered

Telehealth assessments conducted in the studies included in this review at times required clinicians to alter their assessment methods from those typically used during face-to-face consultations. Self-resisted strength assessments, using furniture to assist with the assessment of the shoulder, and educating and utilising another health care provider to assist with the assessments were all techniques used to facilitate the telehealth assessments. Some studies provided resources to participants prior to the telehealth session, which assisted them to understand the assessment process and technologies used.

Some of the difficulties reported in the included studies centred around communication [71], and specifically the explanation and comprehension of information, for example describing pain behavior and location if non-verbal communication was not able to be observed during telephone consultations [54,55,66,72]. Some studies also found that participants were more reserved in their conversation when assessment was conducted over telehealth, or that participants did not understand etiquette required for a video conference consultation [71]. Others reported difficulties in observing posture, as some participants were not comfortable de-robing during the telehealth [74] consultation for a variety of cultural and personal reasons. When working with diverse community groups, Pollard and colleagues [83] suggests we must consider the cultural and ethical needs of individuals, which requires planning, flexibility and sensitivity. As we move forward with utilising synchronous telehealth methods to perform physiotherapy assessments in clinical contexts, we must consider the validity and reliability of the assessment measures used with respect to our clients, and also their presenting condition or diagnosis and contextual factors including the environment they are in and available resources, individual needs and cultural beliefs [83]. These are important elements to consider when deciding if telehealth is appropriate for clients, or if another method of assessment and service delivery is appropriate.

#### Economic impact

There were two studies included in this review that investigated the economic effectiveness of conducting telehealth assessments compared to face-to-face or usual care [46,55]. These concluded that telehealth assessments were slightly more cost-effective when compared with face-to-face care, especially when services were fully utilised, and calculations considered the impacts of waiting times and their effects on an individual’s quality of life. There have been a number of systematic reviews published that have investigated the cost-effectiveness of telehealth or mobile health interventions across medicine [84,85], and psychiatry [86], with cost-effectiveness varying depending on the service and equipment set-up, reporting methods, patient location, travel requirements and needs of the patient. One study concluded that telehealth is most likely cost-effective for patients located in a rural area who have high travel costs [85], however further rigorous studies are required to determine the economic impact that telehealth can have [86], particularly for physiotherapy services.

### Validity and reliability of telehealth assessments

Eighteen of the studies included within this review investigated the concurrent validity and/or reliability of performing synchronous telehealth physiotherapy assessments. The telehealth assessment tools examined in studies included in the current review appeared to be most valid and reliable if they were observational in nature, with the exception of scar assessments and observations of the spine. Telehealth assessments that required a greater degree of technique were also more complex in nature and more difficult to interpret when conducted by telehealth. For example, MSK special tests and neurodynamic testing were less reliable and valid when completed using telehealth systems than when performed face-to-face. This is not surprising, given the extensive training and experience that physiotherapists possess in manual handling and the value that the sense of ‘feel or touch’ can add to interpretation of assessments [87]. Those assessments that involved the use of questionnaires also appeared to be more valid and reliable than some other types of assessment, when conducted via telehealth. This finding is in line with a previous systematic review which reported good test-retest reliability for questionnaires measuring shoulder function [88]. This is to be expected, as no hands-on contact is required to administer these assessments.

Although there appeared to be good levels of agreement between telehealth and face-to-face assessment findings for many of the assessment types and diagnoses, the impact of one incorrect diagnosis or non-referral on the patient and their health condition must be considered [46]. Client and family centred decision-making must also be considered, where individuals may choose a telehealth assessment based on convenience or travel cost, despite the challenges telehealth may have. The use of telehealth in the absence of other face-to-face forms of physiotherapy assessment may improve health outcomes for clients, particularly those in rural and remote areas or impacted by an infectious disease, as it may mean they are able to access services otherwise unavailable to them.

### Limitations of included studies and the present systematic review

Several limitations of the included studies in this literature review influence the conclusions we can draw about the validity, reliability and broader utility of synchronous forms of telehealth physiotherapy assessments. Many studies used a small number of participants with a variety performing the assessment in simulated telehealth environments. There were a few studies, where telehealth assessments were conducted on a convenience sample of participants not necessarily reflective of the wider community or target group for the assessment type – for example, range of movement assessments performed on a healthy population and the MABC-2 performed on typically developing children. The methodological quality of the papers varied, with some papers not adequately describing the telehealth assessments completed or reporting limited information in their findings. The repeated measures design used for many of the validity and reliability studies included in this review has the potential to create a learning effect, with results also influenced by the irritability of the client’s condition. The use of a randomised order of testing or simultaneous testing in most studies helped to counteract this.

The experience of clinicians undertaking the telehealth assessments varied, with reports of higher rates of intra-rater reliability for more experienced clinicians [61]. Levels of training and experience with telehealth systems also varied and should be considered when interpreting results for use in the clinical practice setting. Less experienced clinicians may not achieve the same level of validity and reliability outlined in studies within this review. Telehealth, however, can foster further professional development of less experienced clinicians by enabling more experienced and specialised clinicians to join physiotherapy assessments from anywhere using telehealth, provided they have a stable internet connection and an appropriate device. This ‘peer learning’ was observed in the utility study by Cottrell and colleagues [46], however further studies investigating the validity and reliability of this method of assessment would be beneficial across a broad range of physiotherapy practice areas. Whilst some studies utilised experienced clinicians, some of these were not experienced in the telehealth assessment process. Training in telehealth use is suggested to improve clinician competence, knowledge and acceptance, leading to better skills in using the technology and a higher likelihood of utilising it with clients when suitable [89]. This is important to consider when developing further studies that investigate the use of synchronous telehealth physiotherapy assessments and when using it in a clinical setting.

This systematic review only included studies investigating assessments conducted by a physiotherapist or supervised physiotherapy student. Studies involving assessments conducted by other health professionals, including occupational therapists and medical practitioners, that may also be conducted by physiotherapists were excluded.

Due to the large heterogeneity of the articles that were included in this review and the assortment of analysis used to derive their outcomes, a meta-analysis was not able to be undertaken and therefore, a traditional risk of bias assessment was not conducted. Instead, risk of bias was carefully considered through the critical appraisals performed. Given this was an exploratory study, several case studies/series were included in the results of this systematic review. We acknowledge case studies are considered a low level of evidence and caution needs to be used when interpreting their findings. Some practical information provided through the collection of these case studies can, however, be useful in clinical practice, for example the practicalities of completing a telehealth assessment, including the time it takes to conduct an assessment and the immediate responses of health services during the COVID-19 pandemic.

This systematic review only investigated the validity and reliability of assessments conducted via synchronous forms of telehealth. Asynchronous forms of telehealth such as the use of mobile apps, websites, monitoring devices (eg, smart watches, images and recorded video that are stored and forwarded on to the health care practitioner) are also used within the clinical practice setting. These areas of physiotherapy assessment via telehealth require further investigation and analysis to determine their effectiveness in clinical practice.

## CONCLUSION

Many types of physiotherapy assessments performed using synchronous forms of telehealth appear to be valid and reliable when compared to face-to-face assessments, including assessments of range of movement, muscle strength and endurance, pain intensity, special orthopaedic tests of the shoulder and elbow, Berg Balance Scale (total score), TUG, timed stance test, 6MWT, steps in 360-degree turn, MABC-2 (within 3 points), step test, ABILHAND assessment, active straight leg raise, and circumferential measures of the upper limb. However, most of the research to date on use of telehealth for physiotherapy assessments has been conducted in the MSK field of physiotherapy. Given the recent shift towards this method of service delivery throughout the globe, more research is needed across all areas of physiotherapy. Evidence of validity, reliability and utility of assessments across other areas of physiotherapy practice is emerging but more research with larger sample sizes investigating synchronous telehealth physiotherapy assessments performed in real-world contexts, on readily available technologies and for a range of impairments, is required.

Acceptability of physiotherapy assessments across synchronous methods of telehealth is variable with most still preferring a face-to-face assessment if available. For those requiring telehealth services due to a lack of access to suitable services in their region, satisfaction is high. Telehealth has been vital in maintaining service provision throughout the COVID-19 pandemic however future use by physiotherapists must be decided with consideration given to utilisation of correct and accessible technology and contextual factors such as environmental variables, service access and individual needs and preferences. Telehealth has the potential to limit service access issues, however further research is required to determine which populations would benefit most.

### Contribution of the review

This paper provides a comprehensive synthesis and review of the current research investigating the validity, reliability and utility of performing physiotherapy assessments using synchronous forms of telehealth across all areas of physiotherapy practice. The findings of this review are highly relevant to practitioners and researchers who are providing and/or conducting research in physiotherapy telehealth practice across the globe.

## Additional material


Online Supplementary Document
