# Supplementary material for: The utility of physiotherapy assessments delivered by telehealth: A systematic review
Source: J Glob Health. 2021 Dec 18;11:04072. doi: 10.7189/jogh.11.04072 (PMC8684795; doi:10.7189/jogh.11.04072)
Supplement: Online Supplementary Document [file jogh-11-04072-s001.pdf]

Online Supplementary Document: The Utility of Physiotherapy Assessments Delivered by Telehealth: A Systematic Review

Table S1: PRISMA 2020 Checklist [1]

Section and Topic	Item #	Checklist item	Location where item is reported
**TITLE**			
Title	1	Identify the report as a systematic review.	Yes – report identified as a systematic review in the title
**ABSTRACT**			
Abstract	2	See the PRISMA 2020 for Abstracts checklist.	Yes – abstract checklist completed
**INTRODUCTION**			
Rationale	3	Describe the rationale for the review in the context of existing knowledge.	Yes – reported throughout the *'Introduction'*
Objectives	4	Provide an explicit statement of the objective(s) or question(s) the review addresses.	Yes – provided in the final paragraph of the *'Introduction'*
**METHODS**			
Eligibility criteria	5	Specify the inclusion and exclusion criteria for the review and how studies were grouped for the syntheses.	Yes – Reported in the *'Inclusion and Exclusion Criteria'* and *'Data Extraction, Synthesis and Analysis'* sections of the methods
Information sources	6	Specify all databases, registers, websites, organisations, reference lists and other sources searched or consulted to identify studies. Specify the date when each source was last searched or consulted.	Yes – reported in the *'Search Strategy'* section of the methods
Search strategy	7	Present the full search strategies for all databases, registers and websites, including any filters and limits used.	Yes – reported in the *'Search Strategy'* section of the methods
Selection process	8	Specify the methods used to decide whether a study met the inclusion criteria of the review, including how many reviewers screened each record and each report retrieved, whether they worked independently, and if applicable, details of automation tools used in the process.	Yes – reported in the *'Study Selection'* section of the methods
Data collection process	9	Specify the methods used to collect data from reports, including how many reviewers collected data from each report, whether they worked independently, any processes for obtaining or confirming data from study investigators, and if applicable, details of automation tools used in the process.	Yes – reported in the '*Inclusion and Exclusion Criteria*', *'Quality Assessment'* and *'Data Extraction, Synthesis and Analysis'* sections of the methods
Data items	10a	List and define all outcomes for which data were sought. Specify whether all results that were compatible with each outcome domain in each study were sought (e.g., for all measures, time points, analyses), and if not, the methods used to decide which results to collect.	Yes – reported in the *'Data Extraction, Synthesis and Analysis'* section of the methods
	10b	List and define all other variables for which data were sought (e.g., participant and intervention characteristics, funding sources). Describe any assumptions made about any missing or unclear information.	Yes – reported in the *'Data Extraction, Synthesis and Analysis'* section of the methods
Study risk of bias assessment	11	Specify the methods used to assess risk of bias in the included studies, including details of the tool(s) used, how many reviewers assessed each study and whether they worked independently, and if applicable, details of automation tools used in the process.	Yes – reported in the *'Quality Assessment'* section of the methods.
Effect measures	12	Specify for each outcome the effect measure(s) (e.g., risk ratio, mean difference) used in the synthesis or presentation of results.	Yes – reported in the *'Data Extraction, Synthesis and Analysis'* section of the methods
Synthesis methods	13a	Describe the processes used to decide which studies were eligible for each synthesis (e.g. tabulating the study intervention characteristics and comparing against the planned groups for each synthesis (item #5)).	Yes – reported in the *'Inclusion and Exclusion Criteria'* and *'Study Selection'* section of the methods

Section and Topic	Item #	Checklist item	Location where item is reported
	13b	Describe any methods required to prepare the data for presentation or synthesis, such as handling of missing summary statistics, or data conversions.	Yes – reported in the *'Inclusion and Exclusion Criteria'* section of the methods
	13c	Describe any methods used to tabulate or visually display results of individual studies and syntheses.	Yes – reported in the *'Data Extraction, Synthesis and Analysis'* section of the methods
	13d	Describe any methods used to synthesize results and provide a rationale for the choice(s). If meta-analysis was performed, describe the model(s), method(s) to identify the presence and extent of statistical heterogeneity, and software package(s) used.	Yes – reported in the *'Data Extraction, Synthesis and Analysis'* section of the methods
	13e	Describe any methods used to explore possible causes of heterogeneity among study results (e.g., subgroup analysis, meta-regression).	Yes – reported in the *'Data Extraction, Synthesis and Analysis'* section of the methods
	13f	Describe any sensitivity analyses conducted to assess robustness of the synthesized results.	N/A - In the qualitative synthesis that was conducted, a sensitivity analysis was not performed, however included studies providing only low level evidence, such as case studies, and those of poor methodological quality were reported as such and this is noted in the *'Quality Assessment'* section of the methods
Reporting bias assessment	14	Describe any methods used to assess risk of bias due to missing results in a synthesis (arising from reporting biases).	N/A – as a meta-analysis was not conducted and the synthesis was qualitative in nature, funnel plots were not constructed. Other methods of assessing risk of reporting biases were also not employed.
Certainty assessment	15	Describe any methods used to assess certainty (or confidence) in the body of evidence for an outcome.	Yes – reported in the *'Quality Assessment'* section of the methods.
**RESULTS**			
Study selection	16a	Describe the results of the search and selection process, from the number of records identified in the search to the number of studies included in the review, ideally using a flow diagram.	Yes – reported in Figure 1
	16b	Cite studies that might appear to meet the inclusion criteria, but which were excluded, and explain why they were excluded.	Yes – reported in Figure 1
Study characteristics	17	Cite each included study and present its characteristics.	Yes – reported in Tables 4 and 5
Risk of bias in studies	18	Present assessments of risk of bias for each included study.	Yes – reported in Table 5 and further reported in the Supplementary Material Critical Appraisal Tables
Results of individual studies	19	For all outcomes, present, for each study: (a) summary statistics for each group (where appropriate) and (b) an effect estimate and its precision (e.g. confidence/credible interval), ideally using structured tables or plots.	Yes – reported in Tables 4 and 5
Results of syntheses	20a	For each synthesis, briefly summarise the characteristics and risk of bias among contributing studies.	Yes – reported in the results section and Tables 4 and 5
	20b	Present results of all statistical syntheses conducted. If meta-analysis was done, present for each the summary estimate and its precision (e.g., confidence/credible interval) and measures of statistical heterogeneity. If comparing groups, describe the direction of the effect.	N/A – no meta-analysis or statistical synthesis was conducted. Information is reported in Table 5 regarding relevant statistical estimates and their precision, as reported in the included studies.
	20c	Present results of all investigations of possible causes of heterogeneity among study results.	Yes – reported in Tables 4 and 5 and in the first 4 subsections of the results
	20d	Present results of all sensitivity analyses conducted to assess the robustness of the synthesized results.	N/A - In the qualitative synthesis that was conducted, a sensitivity

Section and Topic	Item #	Checklist item	Location where item is reported
			analysis was not performed, however included studies providing only low level evidence, such as case studies, and those of poor methodological quality were reported as such in Table 4 and Table 5 and in the Supplementary Material Critical Appraisal Tables.
Reporting biases	21	Present assessments of risk of bias due to missing results (arising from reporting biases) for each synthesis assessed.	N/A – as a meta-analysis was not conducted and the synthesis was qualitative in nature, funnel plots were not constructed. Other methods of assessing risk of reporting biases were also not employed.
Certainty of evidence	22	Present assessments of certainty (or confidence) in the body of evidence for each outcome assessed.	Yes – reported in Table 5 and the *'Limitations of Included Studies and the Present Systematic Review'* section of the discussion.
**DISCUSSION**			
Discussion	23a	Provide a general interpretation of the results in the context of other evidence.	Yes – reported in subcategories throughout the *'Discussion'* section of the review
	23b	Discuss any limitations of the evidence included in the review.	Yes – reported in the *'Limitations of Included Studies and the Present Systematic Review'* section of the discussion
	23c	Discuss any limitations of the review processes used.	Yes – reported in the *'Limitations of Included Studies and the Present Systematic Review'* section of the discussion
	23d	Discuss implications of the results for practice, policy, and future research.	Yes – reported in the *'Conclusion'* and *'Contribution of Paper'* sections of the review
**OTHER INFORMATION**			
Registration and protocol	24a	Provide registration information for the review, including register name and registration number, or state that the review was not registered.	Yes – reported under *'Systematic Review Registration'* section
	24b	Indicate where the review protocol can be accessed, or state that a protocol was not prepared.	Yes – reported in the *'Systematic Review'* section of the methods
	24c	Describe and explain any amendments to information provided at registration or in the protocol.	Yes – reported in the *'Deviations for the Protocol'* section of the methods
Support	25	Describe sources of financial or non-financial support for the review, and the role of the funders or sponsors in the review.	Yes – reported in the *'Funding'* section following the Conclusion of the review
Competing interests	26	Declare any competing interests of review authors.	Yes – reported in the *'Conflict of Interest'* and '*Declarations of Interest'* section following the Conclusion of the review
Availability of data, code and other materials	27	Report which of the following are publicly available and where they can be found: template data collection forms; data extracted from included studies; data used for all analyses; analytic code; any other materials used in the review.	Yes – reported in the *'Results'* section of the review and found in the *'Supplementary Material'*

Table S2: Quality Appraisal of Included Validity and Reliability Studies Using the Brink Critical Appraisal Tool [2]

First Author Surname, Year	Title	Study Type	Quality Rating (%)	Quality Rating (Descriptor)
Avelino, 2020	Validation of the telephone-based application of the ABILHAND for assessment of manual ability after stroke	Validity	67%	Good
Cabana, 2010	Interrater agreement between telerehabilitation and face-to-face clinical outcome measurements for total knee arthroplasty	Interrater Agreement	63%	Good
Cottrell, 2018	Agreement between telehealth and in-person assessment of patients with chronic musculoskeletal conditions presenting to an advanced-practice physiotherapy screening clinic	Interrater agreement	88%	Very good
Cox, 2013	Assessing exercise capacity using telehealth: a feasibility study in adults with cystic fibrosis	Validity and feasibility	75%	Good
Galiano-Castillo, 2014	Agreement between telerehabilitation involving caregivers and face-to-face clinical assessment of lymphedema in breast cancer survivors	Validity and reliability	100%	Very good
Hwang, 2017	Assessing functional exercise capacity using telehealth: is it valid and reliable in patients with chronic heart failure?	Validity and reliability	69%	Good
Lade, 2012	Validity and reliability of the assessment and diagnosis of musculoskeletal elbow disorders using telerehabilitation	Validity and inter/intra-rater reliability	69%	Good
Mani, 2019	Concurrent validity and reliability of telerehabilitation-based physiotherapy assessment of cervical spine in adults with non-specific neck pain	Validity and inter/intra-rater reliability	77%	Good
Mehta, 2020	Virtual assessments of knee and wrist joint range motion have comparable reliability with face-to-face assessments	Inter/intra-rater reliability	78%	Good
Nicola, 2018	The feasibility and concurrent validity of performing the Movement Assessment Battery for Children - 2nd Edition via telerehabilitation technology	Validity and feasibility	100%	Very good
Palacin-Marin, 2013	Agreement between telerehabilitation and face-to-face clinical outcome assessments for low back pain in primary care	Validity and inter/intra-rater reliability	85%	Very good
Peterson, 2018	Use of a modified treatment-based classification system for subgrouping patients with low back pain: agreement between telerehabilitation and face-to-face assessments	Interrater agreement	83%	Very good
Richardson, 2017	Physiotherapy assessment and diagnosis of musculoskeletal disorders of the knee via telerehabilitation	Validity and inter/intra-rater reliability	62%	Good
Russell, 2010a	The diagnostic accuracy of telerehabilitation for non-articular lower-limb musculoskeletal disorders	Validity and inter/intra-rater reliability	85%	Very good
Russell, 2013	Internet-based physical assessment of people with Parkinson disease is accurate and reliable: a pilot study	Validity and inter/intra-rater reliability	69%	Good
Russell, 2010b	Telerehabilitation mediated physiotherapy assessment of ankle disorders	Validity and inter/intra-rater reliability	77%	Good
Steele, 2012	Assessment and diagnosis of musculoskeletal shoulder disorders over the internet	Validity and inter/intra-rater reliability	92%	Very good
Truter, 2014	The validity of physical therapy assessment of low back pain via telerehabilitation in a clinical setting	Validity	100%	Very good

The Brink Critical Appraisal Tool [2]

1. If human subjects were used, did the authors give a detailed description of the sample of subjects used to perform the (index) test on?
2. Did the authors clarify the qualification, or competence of the rater(s) who performed the (index) test?
3. Was the reference standard explained?
4. If inter-rater reliability was tested, were raters blinded to the findings of the other raters?
5. If intra-rater reliability was tested, were raters blinded to their own findings of the test under evaluation?
6. Was the order of examination varied?
7. If human subjects were used, was the time period between the reference standard and the index test short enough to be reasonably sure that the target condition did not change between the two tests?
8. Was the stability (or theoretical stability) of the variable being measured taken into account when determining the suitability of the time interval between repeated measures?
9. Was the reference standard independent of the index test?
10. Was the execution of the (index) test described in sufficient detail to permit replication of the test?
11. Was the execution of the reference standard described in sufficient detail to permit its replication?
12. Were withdrawals from the study explained?
13. Were the statistical methods appropriate for the purpose of the study?

Table S3: Quality Appraisal of Included Utility Studies Using the Mixed Methods Appraisal Tool [3]

Author, Year	Title	Quality Appraisal Criteria	Result	Quality Rating (%)	Quality Rating (Descriptor)
Cary, 2016	Benefits and challenges of delivering tele-rehabilitation services to rural veterans	1.1<br>1.2<br>1.3<br>1.4<br>1.5	Yes<br>No<br>Can't tell<br>Can't tell<br>Can't tell	20%	Poor
Cottrell, 2021	Comparing fly-in fly-out and telehealth models for delivering advanced-practice physiotherapy services in regional Queensland: An audit of outcomes and costs	3.1<br>3.2<br>3.3<br>3.4<br>3.5	Yes<br>Yes<br>Yes<br>Can't tell<br>Yes	80%	Very good
Demmelmaier, 2010	Physiotherapists' telephone consultations regarding back pain: a method to analyse screening of risk factors	4.1<br>4.2<br>4.3<br>4.4<br>4.5	No<br>Yes<br>No<br>No<br>Yes	40%	Moderate
Eannucci, 2020	Patient satisfaction for telehealth physical therapy services was comparable to that of in-person services during the COVID-19 pandemic	4.1<br>4.2<br>4.3<br>4.4<br>4.5	Yes<br>Yes<br>Can't tell<br>No<br>Yes	60%	Good
Funderskov, 2019	Telemedicine in specialised palliative care: Healthcare professionals' and their perspectives in video consultations – A qualitative study	1.1<br>1.2<br>1.3<br>1.4<br>1.5	Yes<br>Yes<br>Yes<br>Can't tell<br>Yes	80%	Very good
Harland, 2017	Physiotherapists and general practitioners' attitudes towards 'Physio Direct' phone based musculoskeletal physiotherapy services: a national survey	1.1<br>1.2<br>1.3<br>1.4<br>1.5	Yes<br>Yes<br>Yes<br>Yes<br>Yes	100%	Very good
Hollinghurst, 2013	A pragmatic randomised controlled trial of 'PhysioDirect' telephone assessment and advice services for patients with musculoskeletal problems: economic evaluation	2.1<br>2.2<br>2.3<br>2.4<br>2.5	Yes<br>Can't tell<br>No<br>No<br>No	20%	Poor
Lovo, 2020	Experience of patients and practitioners with a team and technology approach to chronic back disorder management	1.1<br>1.2<br>1.3<br>1.4<br>1.5	Yes<br>Yes<br>Yes<br>Yes<br>Yes	100%	Very good
Mukaino, 2020	An affordable, user-friendly telerehabilitation system assembled using existing technologies for individuals isolated with COVID-19: Development and feasibility study	4.1<br>4.2<br>4.3<br>4.4<br>4.5	Yes<br>Yes<br>No<br>Can't tell<br>No	40%	Moderate
Pearson, 2013	Acceptability to patients of PhysioDirect telephone advice and treatment services: a qualitative investigation	1.1<br>1.2<br>1.3<br>1.4<br>1.5	Yes<br>Yes<br>Can't tell<br>Can't tell<br>Yes	60%	Good
Salisbury, 2013	Effectiveness of PhysioDirect telephone assessment and advice services for patients with musculoskeletal problems: pragmatic randomised controlled trial	2.1<br>2.2<br>2.3<br>2.4<br>2.5	Yes<br>Yes<br>Yes<br>Can't tell<br>Yes	80%	Very good
Wood, 2017	Telehealth clinics increase access to care for adults with cystic fibrosis living in rural and remote Western Australia	4.1<br>4.2<br>4.3<br>4.4<br>4.5	Yes<br>Yes<br>Yes<br>Yes<br>Yes	100%	Very good

Mixed Methods Appraisal Tool (MMAT) Criteria Questions [3]

Screening questions:	S1. Are there clear research questions?<br>S2. Do the collected data allow to address the research questions?
1. Qualitative	1.1 Is the qualitative approach appropriate to answer the research question?<br>1.2 Are the qualitative data collection methods adequate to address the research question?<br>1.3 Are the findings adequately derived from the data?<br>1.4 Is the interpretation of results sufficiently substantiated by data?<br>1.5 Is there coherence between qualitative data sources, collection, analysis and interpretation?
2. Quantitative RCT's<br>(Randomised control trials)	2.1 Is randomization appropriately performed?<br>2.2 Are the groups comparable at baseline?<br>2.3 Are there complete outcome data?<br>2.4 Are outcome assessors blinded to the intervention provided?<br>2.5 Did the participants adhere to the assigned intervention?
3. Quantitative non-randomized	3.1 Are the participants representative of the target population?<br>3.2 Are measurements appropriate regarding both the outcome and intervention (or exposure)?<br>3.3 Are there complete outcome data?<br>3.4 Are the confounders accounted for in the design and analysis?<br>3.5 During the study period, is the intervention administered (or exposure occurred) as intended?
4. Quantitative descriptive	4.1 Is the sampling strategy relevant to address the research question?<br>4.2 Is the sample representative of the target population?<br>4.3 Are the measurements appropriate?<br>4.4 Is the risk of nonresponse bias low?<br>4.5 Is the statistical analysis appropriate to answer the research question?
5. Mixed Methods	5.1 Is there an adequate rationale for using a mixed methods design to address the research question?<br>5.2 Are the different components of the study effectively integrated to answer the research question?<br>5.3 Are the outputs of the integration of qualitative and quantitative components adequately interpreted?<br>5.4 Are divergences and inconsistencies between quantitative and qualitative results adequately addressed?<br>5.5 Do the different components of the study adhere to the quality criteria of each tradition of the methods involved?

References

1 Page MJ, McKenzie JE, Bossuyt PM, Boutron I, Hoffmann TC, Mulrow CD, et al. The PRISMA 2020 statement: An updated guideline for reporting systematic reviews. Bmj. 2021;372:n71.

2 Brink Y, Louw QA. Clinical instruments: Reliability and validity critical appraisal. J Eval Clin Pract. 2012;18:1126-32.

3 Hong QN, Pluye P, Fabregues S, Bartlett G, Boardman F, Cargo M, et al. Mixed Methods Appraisal Tool (MMAT) version 2018. 2018.
